# Controls on planktonic foraminifera apparent calcification depths for the northern equatorial Indian Ocean

**DOI:** 10.1371/journal.pone.0222299

**Published:** 2019-09-12

**Authors:** Stephanie Stainbank, Dick Kroon, Andres Rüggeberg, Jacek Raddatz, Erica S. de Leau, Manlin Zhang, Silvia Spezzaferri

**Affiliations:** 1 Department of Geosciences, University of Fribourg, Fribourg, Switzerland; 2 School of GeoSciences, Grant Institute, University of Edinburgh, Edinburgh, United Kingdom; 3 Institute of Geosciences, Goethe University Frankfurt, Frankfurt am Main, Germany; 4 Frankfurt Isotope and Element Research Center (FIERCE), Goethe University Frankfurt, Frankfurt am Main, Germany; Vrije Universiteit Amsterdam, NETHERLANDS

## Abstract

Within the world’s oceans, regionally distinct ecological niches develop due to differences in water temperature, nutrients, food availability, predation and light intensity. This results in differences in the vertical dispersion of planktonic foraminifera on the global scale. Understanding the controls on these modern-day distributions is important when using these organisms for paleoceanographic reconstructions. As such, this study constrains modern depth habitats for the northern equatorial Indian Ocean, for 14 planktonic foraminiferal species (*G*. *ruber*, *G*. *elongatus*, *G*. *pyramidalis*, *G*. *rubescens*, *T*. *sacculifer*, *G*. *siphonifera*, *G*. *glutinata*, *N*. *dutertrei*, *G*. *bulloides*, *G*. *ungulata*, *P*. *obliquiloculata*, *G*. *menardii*, *G*. *hexagonus*, *G*. *scitula*) using stable isotopic signatures (δ^18^O and δ^13^C) and Mg/Ca ratios. We evaluate two aspects of inferred depth habitats: (1) the significance of the apparent calcification depth (ACD) calculation method/equations and (2) regional species-specific ACD controls. Through a comparison with five global, (sub)tropical studies we found the choice of applied equation and δ^18^O_sw_ significant and an important consideration when comparing with the published literature. The ACDs of the surface mixed layer and thermocline species show a tight clustering between 73–109 m water depth coinciding with the deep chlorophyll maximum (DCM). Furthermore, the ACDs for the sub-thermocline species are positioned relative to secondary peaks in the local primary production. We surmise that food source plays a key role in the relative living depths for the majority of the investigated planktonic foraminifera within this oligotrophic environment of the Maldives and elsewhere in the tropical oceans.

## Introduction

Planktonic foraminifera are protozoans widely used in paleoceanographic and paleoclimatic studies to interpret and track past marine conditions [1]. They occupy surface to sub-thermocline depths in the pelagic ocean with regional differences in food and seawater properties constraining their latitudinal, temporal and depth distributions. Average living depths (ALD)/Apparent calcification depths (ACD) of foraminiferal species are not globally ubiquitous [2,3]. Thus, accurately constraining regional estimates is important as this bears significance when selecting suitable species for paleoceanographic reconstructions and for interpreting the oceans past vertical thermal structure [4–9].

There are various direct (e.g. concentration profiles calculated from multinet plankton tows, opening-closing nets and sediment traps) and indirect (e.g. test/shell geochemical signatures) methods which can be used to denote foraminifera ALDs and ACDs, respectively. Direct methods allow the sampling of living foraminifera *in situ* and at a more refined temporal scale yet, are limited by the practicality of refined depth stratified sampling, low abundances, patchiness and inherently incorporate dead or dying foraminiferal tests (shells) from the settling pelagic rain. On the contrary, indirect methods have their own restrictions, as the bulk geochemical signatures are a collated record of the ambient environmental conditions experienced as these micro-organisms migrate within the water column during their life cycle. According to [1] these organisms have species-specific reproductive depths which are generally associated with the pycnocline. However, as the majority of the calcite is added towards the end of the foraminiferal ontogenetic cycle, adult specimen’s geochemical signatures are generally weighted by the final few precipitated chambers [2]. Furthermore, numerous studies [10–12] have addressed the significant relationship between the geochemical signatures (e.g. δ^18^O, δ^13^C, Mg/Ca and Sr/Ca) and size of the planktonic foraminiferal calcitic test. The species-specific size ranges selected for measurement are, therefore, important to take into consideration when conducting such geochemical analyses.

Whether using the δ^18^O signatures or Mg/Ca ratios from foraminiferal tests to calculate ACDs, a calibrated temperature equation, based on observations from modern oceans, is required. While calibrated for a specific species, size range, region and temperature range, these equations still require additional parameters, which for the paleo-record are often assumed/calculated and, nonetheless, not always available for the present day (i.e. seawater δ^18^O). Furthermore, compounding factors such as varying cleaning methods (i.e. oxidative with/without reductive cleaning of Mg/Ca samples), post-depositional forces (i.e. diagenesis), species-isotopic offsets, species ecology and lack of regional equations can further influence the calculated estimates [12–15].

**Fig 1:**
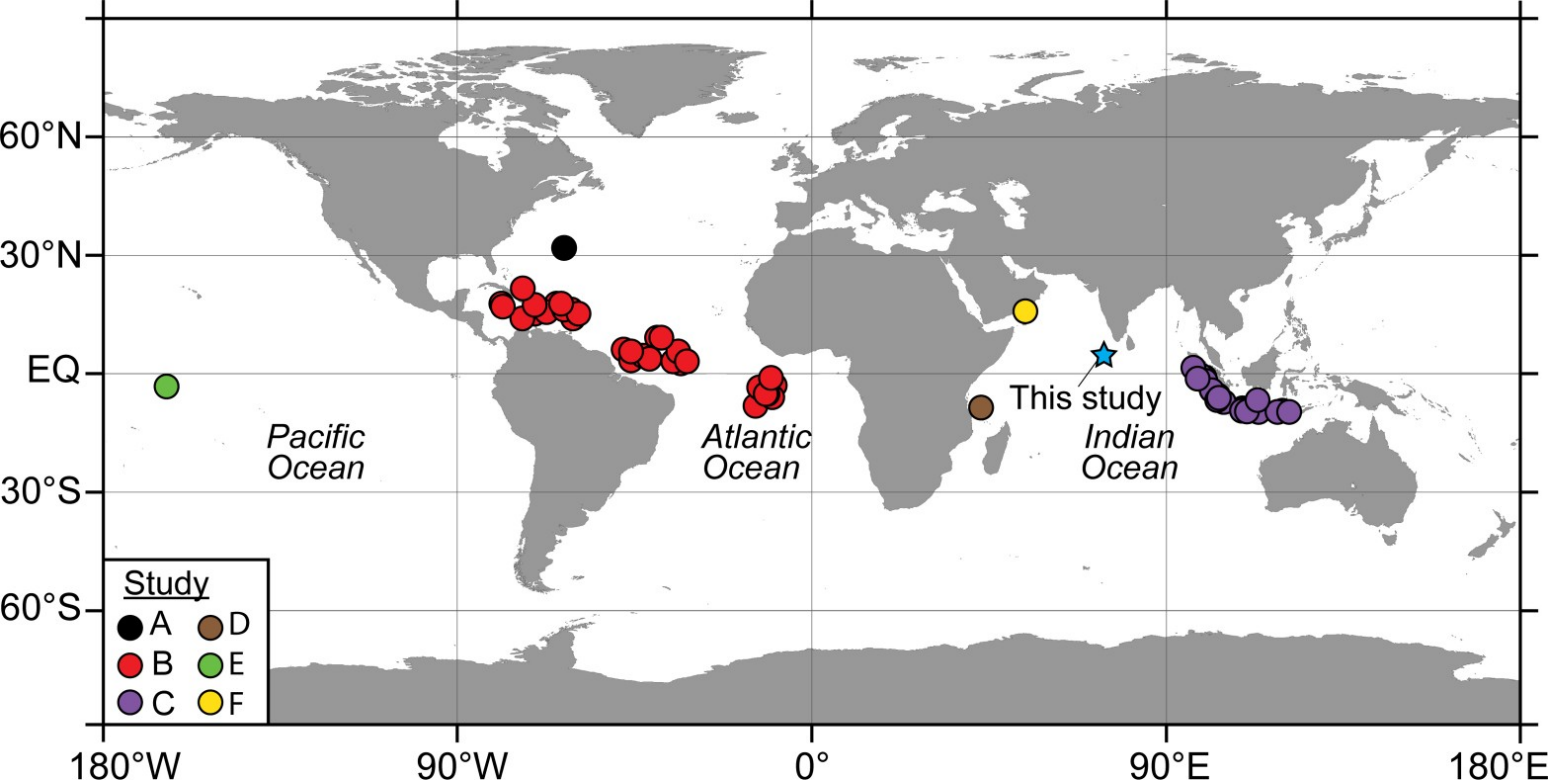
Location of the study site in the northern equatorial Indian Ocean, the Maldives (blue star) and the comparative studies referenced in this work: A[13], B[16], C[14], D[12], E[17] and F[3] (world map from [18]).

Therefore, in this study, we use stable isotopic signatures (δ^18^O) and Mg/Ca ratios to estimate the inferred depth habitats, here referred to as ACDs, of 14 planktonic foraminiferal species from the Maldives in the northern equatorial Indian Ocean (Table 1). We use our geochemical data collected from core top samples, to assess both stable isotope and Mg/Ca ACD calculation methods and associated published equations. We test and compare the methods of five global studies [12–14,16,17] as they represent (sub)tropical regions, similarly to the present study site, yet have different hydrographic and climatic controls and cover the full range of investigated species (Fig 1).

**Table 1:** Species list and associated data for geochemical analysis.

Species	Life strategy	Size-fraction analysed (μm)	Stable isotopes		Mg/Ca
			No. analysed		
*Globigerinoides ruber* (w)	Shallow/ intermediate planktonic	212–250; 355–400	5; 2	✓	✓
*Globigerinoides elongatus*		212–250	5	✓	
*Globigerinoides pyramidalis*		212–250	5	✓	
*Globoturborotalita rubescens* (p)		125–150	24	✓	
*Trilobatus sacculifer* (w/s)		300–355	2	✓	✓
*Globigerinella siphonifera*		300–355	2	✓	✓
*Globigerinita glutinata* (w/b)		125–150	24	✓	
*Neogloboquadrina dutertrei*		355–400	2	✓	
*Globigerina bulloides*		212–250	5	✓	✓
*Globorotalia ungulata*		212–250	5	✓	
*Pulleniatina obliquiloculata* (w/c)		355–400	2	✓	✓
*Globorotalia menardii*		300–355	2	✓	✓
*Globorotaloides hexagonus*	Deep planktonic	180–212; 212–250	9; 5	✓	
*Globorotalia scitula*		125–150; 180–212	24; 9	✓	
*Cibicides wuellerstorfi/mabahethi*	Benthic	>212	3	✓	✓

w = white; p = pink; w/s = with sac; w/b = with bulla; w/c = with cortex

Consequently, using newly acquired core top data from the Maldives two main hypotheses are tested:

1. The choice of ACD calculation method can have a significant impact on the final vertical positioning of individual planktonic foraminiferal species.
2. The ACDs of (sub)tropical planktonic foraminifera are (i) regionally distinct and (ii) controlled by differences in food sources (i.e. position of the deep chlorophyll maximum) linked to thermocline dynamics.

### Regional setting

**Fig 2:**
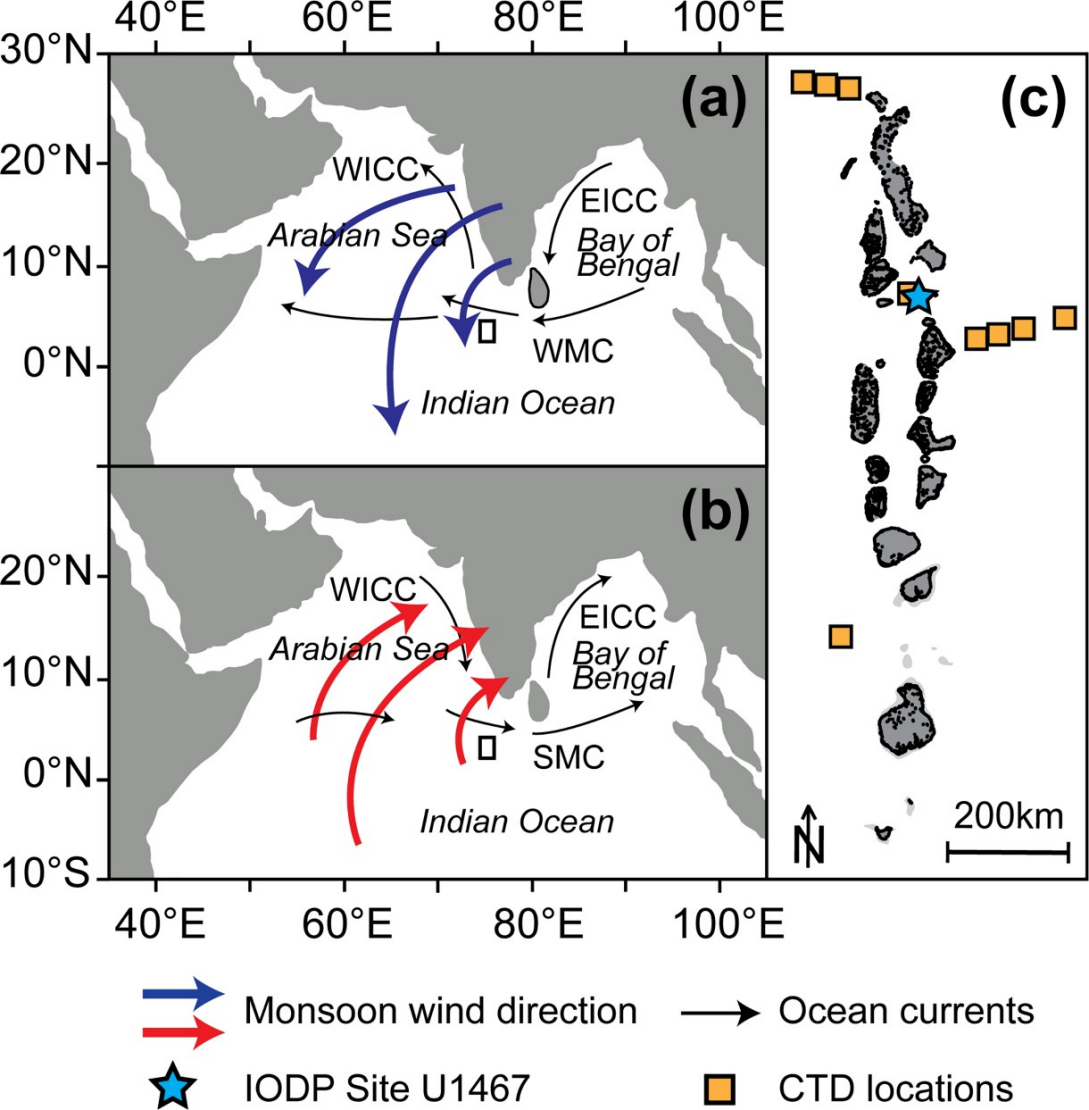
**Maps showing seasonal reversal of the South Asian Monsoon (SAM) winds and associated ocean currents during (a) winter and (b) summer and (c) the location of the study Site U1467 in the Maldives (blue star) and the measurement sites of the CTD profiles used in this study (yellow squares).** WICC = West India Coastal Current, WMC = Winter Monsoon Current; SMC = Summer Monsoon Current; EICC = East India Coastal Current (c. modified after [20]).

The Asian Monsoon system covers a large region from the western Arabian Sea to East Asia, extending down to North Australia [19]. It is a dynamic climatic system affecting both atmospheric circulation and precipitation. The seasonal reversal in the wind system results in warm, wet continental summers and cool, dry continental winters. These seasonal fluctuations also result in changes in the regional ocean current strengths and directions (Fig 2). Furthermore, regional seasonality in sea surface temperatures (SST), salinity and upwelling occurs. The Asian Monsoon is divided into two subsystems, the South Asian (also known as the Indian) Monsoon (SAM or IM) and the East Asian Monsoon (EAM). The two associated subsystems, roughly divided at a longitude of 105°E, are inherently different due to variations in sea-land distributions [19]. The SAM is the predominant climatic system influencing our study area in the Maldives (Fig 2).

**Fig 3:**
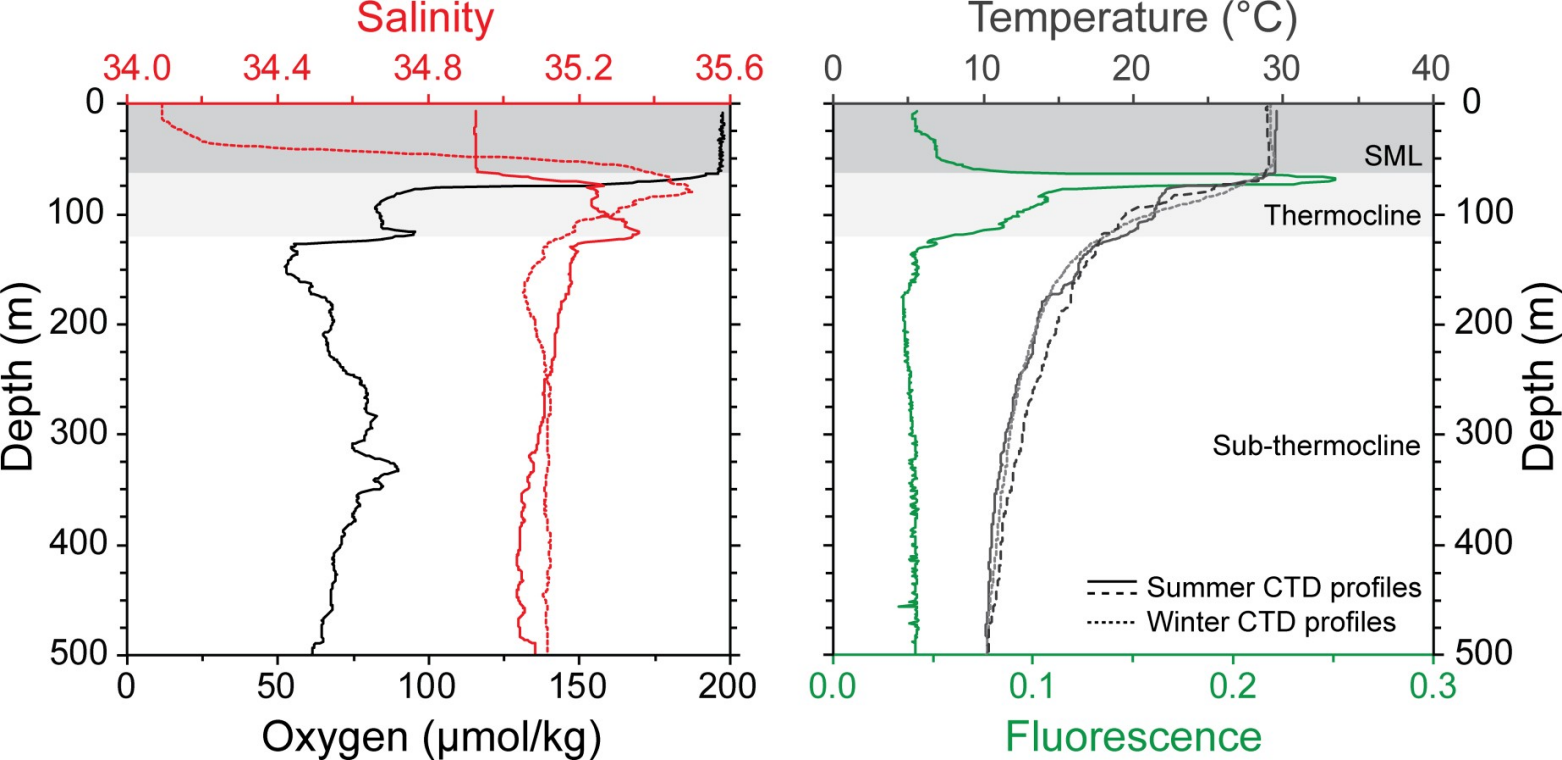
Conductivity, Temperature and Depth (CTD) probe data from the Maldives region, including summer (coarsely dashed and solid lines) and winter (finely dashed line) salinity, temperature, oxygen and fluorescence profiles [24,26,27]. SML = surface mixed layer.

The Maldives archipelago, located south-west of the Indian sub-continent in the northern Indian Ocean, is located within the Arabian Sea. It is a partially drowned carbonate platform, consisting of two rows of North-South orientated atolls bordering an Inner Sea [21]. It sits on a pinnacle, 2000–4000 m, above the adjacent seafloor limiting the maximum depth of the Inner Sea to ~500 m [22]. The SAM-driven winds, northeast and southwest in winter and summer, respectively, drive modern currents in the Maldives (Fig 2). Furthermore, its shallow position results in its intersection with the Oxygen Minimum Zone (OMZ), which regionally extends from ~ 150 m to 1200 m [23]. According to [24] a local oxygen minimum (~41.017 μmol/kg), generated by wind driven upwelling [25] is observed at ~ 500 m water depth, this is similar to regional Conductivity, Temperature and Depth probe (CTD) data recorded marginally south of the Maldives by [26], (Fig 3).

According to [24], temperature stratification is present across the entire Inner Sea. The surface mixed layer (SML) extends down to 60–70 m and has a temperature range between 28 and 29°C [24,26,27]. The fluorescence profile shows that a deep chlorophyll maximum (DCM), peak in primary production, is present at the base of the SML with a primary peak (F1) at ~69 m water depth with two secondary peaks (F2 and F3) extending from ~80–175 m (Fig 3; [24,26,27]). A sharp thermocline is present at ~70–120 m water depth with temperatures decreasing rapidly across the thermocline down to ~10.21°C at 500 m (Fig 3). Limited seasonal differences occur in the temperature profiles. On the contrary, the seasonal salinity profiles show marked differences, which for the most part are restricted to the SML with the maximum peak at 75–80 m (Fig 3). During the summer southwest monsoon, strong winds generate the eastward flowing Summer Monsoon Current (SMC) from June to October (Fig 2). The reversal in the currents, during the winter northeast monsoon, results in an influx of low salinity water transported from the Bay of Bengal into the southeastern Arabian Sea by the Winter Monsoon Current (WMC) from December to April [28]. As such, the surface winter-summer salinities differ by 0.80 psu, with lower salinities of ~34.10 psu recorded in winter and higher values of ~ 34.90 psu recorded in summer.

## Materials and methods

**Fig 4:**
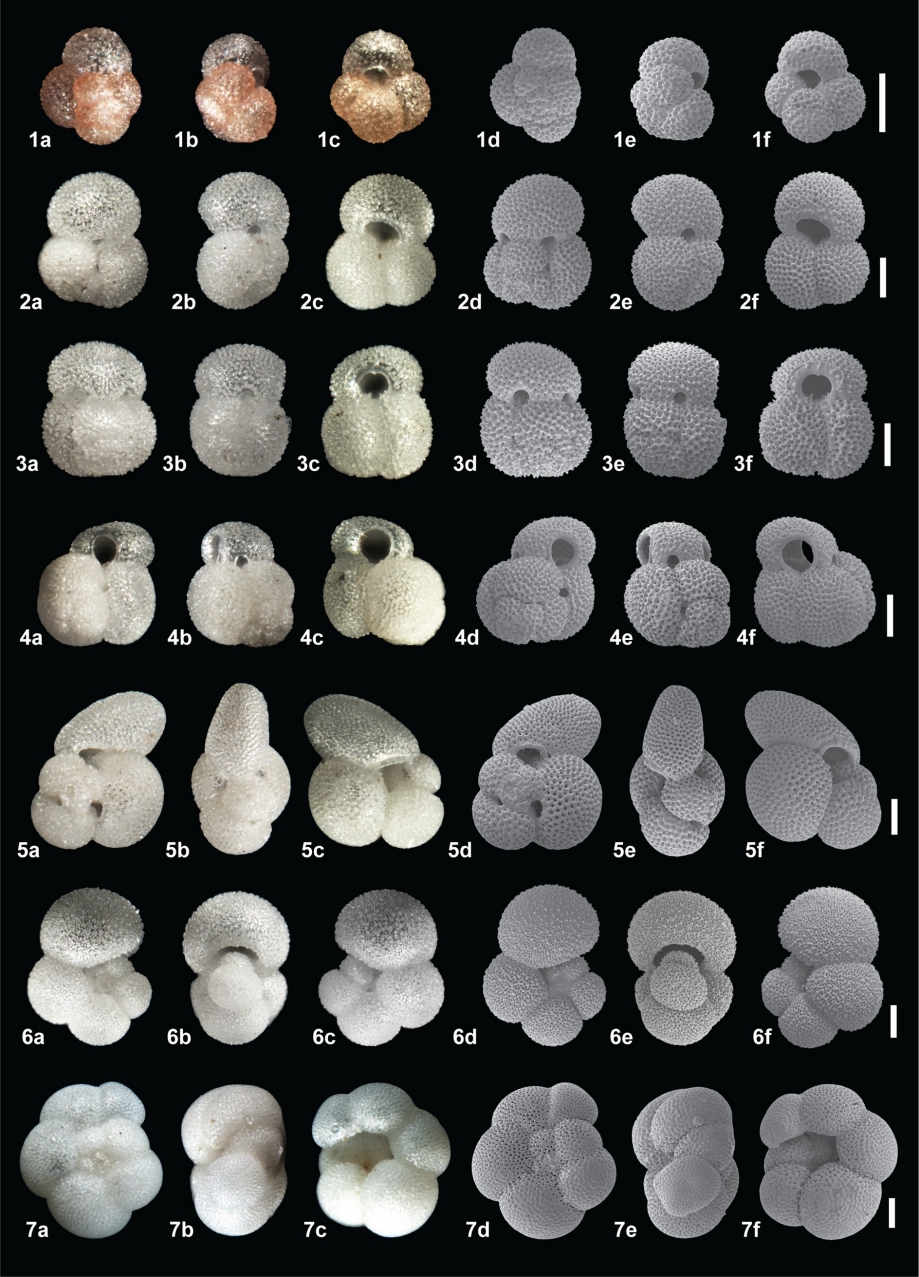
**Plate illustrating light microscope and Scanning Election Microscope (SEM) images of the spiral (a, d), ventral (b, e) and umbilical view (c, f) for 1. *Globoturborotalita rubescens* (p), 2. *Globigerinoides ruber* (w), 3. *Globigerinoides elongatu*s, 4. *Globigerinoides pyramidalis*, 5. *Trilobatus sacculifer* (w/s), 6. *Globigerinella siphonifera*, 7. *Neogloboquadrina dutertrei*.** All scale bars = 100 μm, p = pink, w = white, w/s = with sac.

**Fig 5:**
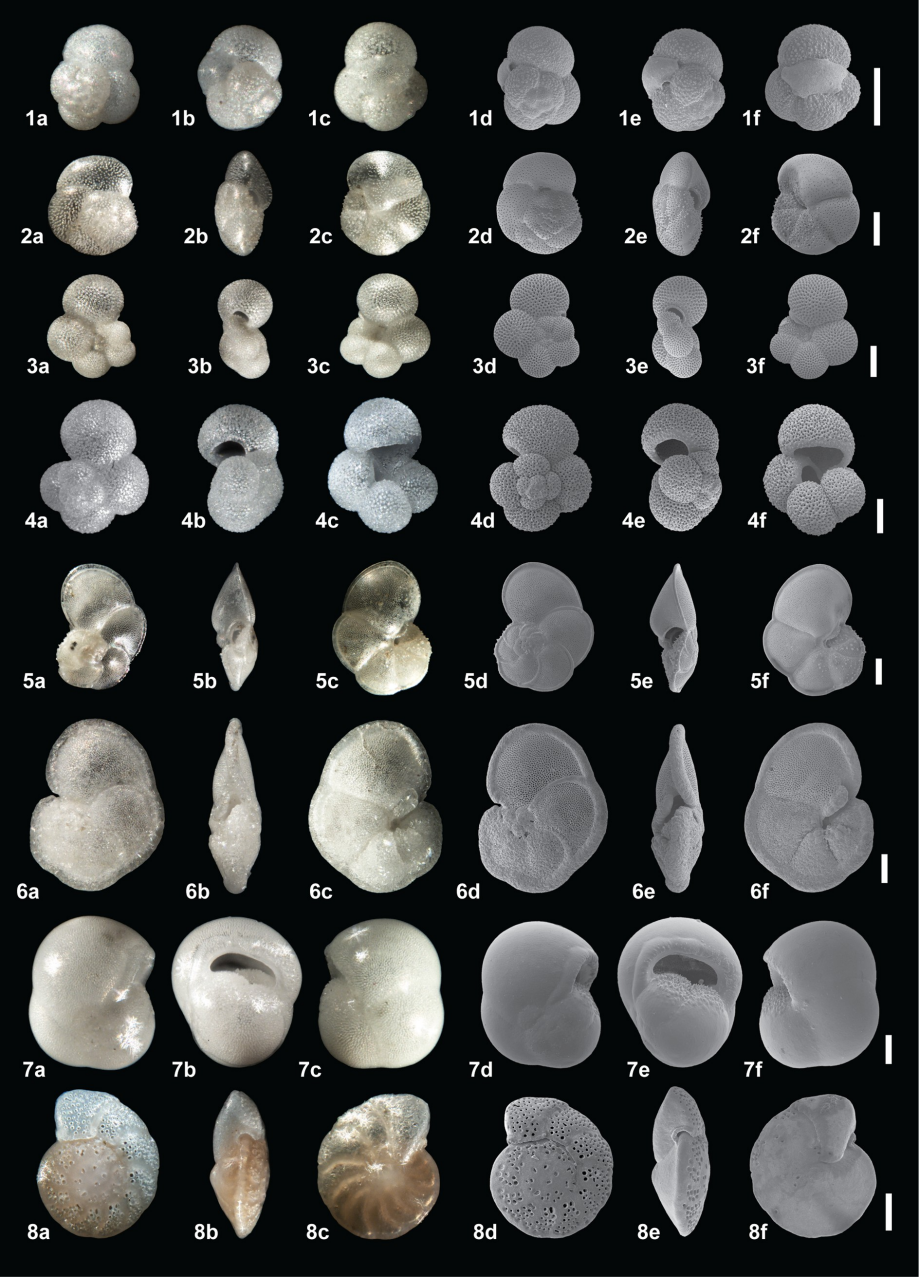
**Plate illustrating light microscope and Scanning Election Microscope (SEM) images of the spiral (a, d), ventral (b, e) and umbilical view (c, f) for 1. *Globigerinita glutinata* (w/b), 2. *Globorotalia scitula*, 3. *Globorotaloides hexagonus*, 4. *Globigerina bulloides*, 5. *Globorotalia ungulata*, 6. *Globorotalia menardii*, 7. *Pulleniatina obliquiloculata* (w/c), 8. *Cibicides mabahethi*.** All scale bars = 100 μm, w/b = with bulla, w/c = with cortex.

We paired stable isotope (δ^18^O and δ^13^C) and select Mg/Ca measurements of 14 planktonic foraminiferal species to calculate their ACDs and infer depth habitat controls (Figs 4 and 5). Planktonic foraminifera live in the upper ~500 m of the ocean [29] and thus species were selected with previously reported depth habitat preferences to span both shallow and deeper waters (i.e. SML, thermocline and sub-thermocline depths), (Table 1, e.g.: [12,16] and references within). Additionally, a benthic representative made up of two *Cibicides* species was included in the analyses. This allowed the bottom water temperatures to be constrained and as modern day CTD profiles are available they were used to validate the applied ACD calculation methods for the planktonic foraminifera (i.e. isotopic vs Mg/Ca ACD calculation methods, explained in further detail in the subsequent sections).

Target foraminifera species were isolated from mudline (sediment/water interface) samples (unlithified foraminifera-rich wackestone to packstone, [30]) of the International Ocean Discovery Program (IODP) Expedition 359, Site U1467 (Fig 2). This site, located at 4°51.0274′N, 73°17.0223′E, was drilled in the middle of the Inner Sea of the Maldives Archipelago at a depth of 487 m [30] within the drift deposit sediments. The mudline samples are deemed modern as portions were stained onboard with Rose Bengal (1 g/L) which verified the presence of living ostracods as well as benthic foraminifera [30]. Samples were air dried, weighed and washed through a 32 μm sieve. After which, they were dried in an oven for 48 hours at 30°C, weighed and dry sieved into discrete fractions for picking (Table 1). All species were picked from restricted size ranges (Table 1). Appropriate species-specific size ranges were selected, based on the published literature (e.g. [10,12,31], in order to limit intra-specific ontogenetic isotopic fractionation effects. Furthermore, two size ranges were measured for three of the targeted species; *G*. *ruber* (w), *G*. *hexagonus* and *G*. *scitula* to evaluate (pre)adult versus juvenile depth preferences. With two recognised morphotypes of *G*. *siphonifera*, we strictly picked the large evolute forms conforming to Type I [1,32] for the geochemical analyses. For all species only pristine ‘glassy’ specimens were picked which had no infilling, discolouration or evidence of reworking (i.e. broken chambers), (Figs 4 and 5).

All 15 species (14 planktonic and 1 benthic, Figs 4 and 5) were analysed for their stable isotopic signatures (δ^18^O and δ^13^C) at the Grant Institute of the University of Edinburgh on a Thermo Electron Delta+ Advantage mass spectrometer integrated with a Kiel carbonate III automated extraction line (Table 1). For the isotopic analysis, only *C*. *mabahethi* was used as the benthic representative (Fig 5). Two to three replicates were measured for each species, with the exception of *C*. *mabahethi* due to the rarity of the species. Prior to analysis, all samples were precleaned by high-powered ultrasonication in Milli-Q water for a few seconds to remove any contaminating phases. The number of specimens analysed varied according to the species and size fraction used, however, in all instances 0.05 mg was required for the analyses (Table 1). The laboratories internal standard was used to calibrate the measurements, which are expressed as parts per mil (‰) relative to VPBD. Replicate measurements give the instrument an analytical precision of 0.1 ‰ for δ^18^O and δ^13^C.

Corresponding Mg/Ca ratios were also measured in tests of seven of the species, over the same size fractions used for the stable isotopic analyses (Table 1). Not all species could be analysed, due to the larger sample size needed for these measurements (±25 specimens), compounded by the low abundances of the rarer species and species-specific target size fraction restrictions. Additionally, replicate measurements were only feasible for four of the target species and due to the limited number of benthic specimens in the mudline sample, *C*. *mabahethi* and *C*. *wuellerstorfi* were combined to obtain Mg/Ca ratios representative of the Inner Sea bottom waters. Prior to measurements, samples were ultrasonically (high-power) cleaned for a few seconds in Milli-Q water. This removed any adhering phases that could introduce sources of carbonate contamination. Subsequently, all samples were cleaned according to the standard oxidative protocol of [33] with the exclusion of the reductive cleaning step due to the small sample sizes [34].

Briefly, samples were cracked (the foraminiferal tests were broken open) and rinsed three times in methanol and Milli-Q water in order to remove adherent clay particles. Next, samples were leached with a very weak 0.001N HNO_3_ acid solution prior to dissolving samples in 0.075M HNO_3_. Analyses were conducted at the Institute of Geosciences of the Goethe-University of Frankfurt by inductively coupled plasma optical emission spectrometry (ICP-OES) Thermoscientific iCap 6300 (dual viewing). The final centrifuged sample solution was diluted with yttrium water (1 mg/l) prior to measurement in order to correct for matrix effects during ICP-OES analyses. Element/Ca measurements were drift-corrected and standardized using an internal consistency standard (ECRM 752–1, 3.761 mmol/mol Mg/Ca, [35]). The reproducibility of the ECRM was ~ 0.1 mmol/mol (2 SD). Furthermore, blanks were routinely run to monitor potential contamination during the cleaning process. During all Mg/Ca measurements the elements Al, Fe, and Mn were screened to check for Mn-Fe oxide coatings and clay mineral contamination.

As replicates were measured for the geochemical data (stable isotopes and Mg/Ca), averages were calculated for all species and used in all subsequent calculations (see results section for the raw geochemical data).

### Apparent calcification depths (ACDs)

In the literature, authors use either one or a combination of isotope or Mg/Ca methods to calculate their foraminifera ACDs. Each method involves user discretion in the selection of available equations and select variables, thus unavoidably a degree of uncertainty is incorporated into the calculated ACDs. We chose five global, low-latitude, studies from different ocean basins to test their choice of method (Method one: isotope ACD calculations and Method two: Mg/Ca ACD calculations) and equations on our dataset [12–14,16,17]. By using this integrated approach, we were able to assess the accuracy of our estimates as well as comment on the strengths and limitations of each method/equation, in order to select the most appropriate to apply in our study. In all instances, the benthic representative was used as a control to constrain the most applicable equations/approach.

Method one: Foraminifera calcite δ^18^O (hereafter referred to as δ^18^O_c_) was utilized in two different ways (Method 1.1 and 1.2) to calculate isotope derived ACDs (hereafter referred to as isotope-ACDs). Firstly, in Method 1.1 sea water temperatures were calculated using measured δ^18^O_c_ values, an assumed seawater δ^18^O (hereafter referred to as δ^18^O_sw_) value and published δ^18^O_c_-temperature equations. ACDs were then assigned with reference to modern CTD temperature profiles [12] (Table 2, Fig 3). On the contrary, Method 1.2 involved rearranging published δ^18^O_c_-temperature equations, inserting δ^18^O_sw_ depth profile data and modern CTD temperature data to calculate the δ^18^O equilibrium (hereafter referred to as δ^18^O_e_) depth profile. The depths at which the measured δ^18^O_c_ values matched the δ^18^O_e_ were allocated as the ACDs [13,14,16,17], (Table 2). Method 1.1 and 1.2 are very similar; however, the most notable difference is the δ^18^O_sw_ allocations. The former method utilizes either just a single δ^18^O_sw_ value for all species (as is commonly done in the literature) or a combination of different values depending on the assumed species living depth (in this study a broad classification of shallow-/intermediate- versus deep-dwellers was used). On the contrary, the latter method uses a complete δ^18^O_sw_ depth profile to calculate incrementally the δ^18^O_e_ values. As the δ^18^O_sw_ allocation is essential in the δ^18^O_c_-temperature equations, this distinction is important and justifies the testing of both Methods 1.1 and 1.2.

**Table 2:** Global studies used in this comparison (Fig 1).

Study	Reference	Study location	Sample type	ACD calculation method
A	[13]	North Atlantic Ocean	Sediment trap	1.2
B	[16]	Atlantic Ocean	Core top	1.2
C	[14]	Eastern Indian Ocean	Core top	1.2
D	[12]	Western Indian Ocean	Box core	1.1
E	[17]	Pacific Ocean	Plankton tows	1.2 & 2

Method one: isotope-ACD calculation methods (1.1: temperature versus 1.2: δ^18^O_e_) and Method two: Mg/Ca-ACD calculation method.

Method two: Measured foraminifera Mg/Ca ratios were used to calculate Mg/Ca derived ACDs (hereafter referred to as Mg/Ca-ACDs). Seawater temperatures were calculated using the Mg/Ca ratios and published species-specific Mg/Ca-temperature equations. ACDs were then assigned with reference to the modern CTD temperature profiles ([13,17]; Table 2, Fig 3).

### Method one: Isotope-ACD calculation methods

A database of species-specific δ^18^O-temperature equations, for the investigated species, was compiled (S1 Table). Four factors were noted for each, including size fraction measured, sample type, geographical location and temperature calibration range. The equations with factors most comparable to our study and study site were selected (S1 Table). It should be noted that species-specific equations were unfortunately not available for all the investigated species.

[12] noted only small differences between calibrations, thus in addition to the application of species-specific equations (similarly to [14]); we tested the implications of using a single equation for all investigated planktonic species. Thus, similarly to the studies of [12], [17], [16] and [13] the *Globigerinoides sacculifer* (now known as *Trilobatus sacculifer* [36]) temperature Eq 1 of [37], the multi-species foraminifera Eq 2 of [38], the inorganic calcite Eq 3 of [39] and the synthetic calcite Eq 4 of [40] were utilized for all species.

$$T({}^{\circ}C)=16.998-4.520(\delta^{18}O_{c}-\delta^{18}O_{sw})+0.028{\left(\delta^{18}O_{c}-\delta^{18}O_{sw}\right)}^{2}$$ (1)

$$T({}^{\circ}C)=14.32-4.28(\delta^{18}O_{c}-\delta^{18}O_{sw})+0.07{\left(\delta^{18}O_{c}-\delta^{18}O_{sw}\right)}^{2}$$ (2)

$$T({}^{\circ}C)=16.10-4.64(\delta^{18}O_{c}-\delta^{18}O_{sw})+0.09{\left(\delta^{18}O_{c}-\delta^{18}O_{sw}\right)}^{2}$$ (3)

$$T({}^{\circ}C)=16.90-4.38(\delta^{18}O_{c}-\delta^{18}O_{sw})+0.10{\left(\delta^{18}O_{c}-\delta^{18}O_{sw}\right)}^{2}$$ (4)

In all instances, the δ^18^O_sw_ values were converted from the VSMOW to VPBD scale by subtracting a suitable correction relative to each equation [41–44].

For Method 1.1, the foraminiferal δ^18^O_c_ values were measured, therefore, the only missing and thus assumed variable for these equations was the δ^18^O_sw_. We, therefore used δ^18^O-temperature Eqs 1–4 in addition to species-specific equations in two consecutive calculations to assess the influence of the assigned δ^18^O_sw_ value.

Firstly, an average δ^18^O_sw_ value of 0.39 ‰ was used for all equations. This was based on *in situ* measured seawater values from 0 m and ±500 m from the Maldives region [45–48]. All calculations were then repeated using regionally calculated δ^18^O_sw_ vertical profiles. Regional δ^18^O_sw_ depth profiles were calculated by rearranging calibrated salinity equations. The salinity Eq 5 of [49], Eq 6 of [47] and Eq 7 of [50] were calibrated for the study region and thus all were initially tested. Due to seasonal differences, in predominantly the SML, salinity CTD profiles from both summer [26] and winter [27] were used in the calculations. Ultimately, published regional δ^18^O_sw_ values for the surface 0 m [45–47] and ±500 m depth [48] and the gridded data set of [51] were used as controls, to select the most suitable equation for the generation of the regional δ^18^O_sw_ depth profile. Subsequently, average δ^18^O_sw_ values for 0–75 m (i.e. 75 m coincides with the salinity maximum) and 75–500 m were calculated and used with the δ^18^O-temperature equations for the reported shallow-intermediate and deeper-dwelling species, respectively (Table 1).

$$\delta^{18}O_{sw}=0.28.S-9.24$$ (5)

$$\delta^{18}O_{sw}=0.57.S-20$$ (6)

$$\delta^{18}O_{sw}=(0.08\pm 0.05).S-(2.41\pm 1.87)$$ (7)

All calculated temperatures were correlated with the modern-day seasonal (summer and winter) CTD profiles and the corresponding depths assigned as the isotope-ACDs. The local temperature profiles of [24,26,27] were all used to account for seasonal variability and as such the final designated isotope-ACDs are averages of the summer and winter calculations.

For Method 1.2, δ^18^O_c_ is assumed to have precipitated in equilibrium with the seawater. The seasonal δ^18^O_e_ vertical profiles were then calculated by rearranging δ^18^O-temperature species-specific equations in addition to Eqs 1–4. All equations were used together with the seasonally calculated δ^18^O_sw_ vertical profiles, obtained using seasonal salinity data [26,27]. The measured planktonic foraminiferal δ^18^O_c_ values were compared with the δ^18^O_e_ profiles to infer their isotope-ACDs and an overall average from the summer and winter data calculated to assign the respective isotope-ACDs.

### Method two: Mg/Ca-ACD calculation method

A database of published Mg/Ca-temperature equations was compiled for all investigated species (S2 Table) with five main factors noted for each (i.e. size fraction measured, sample type, geographical location, temperature calibration range and cleaning method used). The nature of the exponential function combined with the range in associated factors can result in widespread calculated temperatures for each species. Additionally, due to reasons explained in the methods section above, Mg/Ca ratios were only measured for seven of the target species. Consequently, this approach was not used as a principal ACD calculation method but instead to validate the assigned isotope-ACDs.

For all species, there are numerous published equations. To more objectively select the most appropriate species-specific equations for our study location, the following steps were taken:

1. Equations calibrated using reductively cleaned individuals were treated with caution as several authors [33,52,53] have shown this step reduces the Mg/Ca ratios of both planktonic and benthic species. [33] reported the reductive treatment resulted in a 10–15% reduction in the Mg/Ca for planktonic foraminifera whereas [52] reported a reduction of 0.2 mmol/mol for the benthic *Cibici(doi)des*.
2. An upper temperature limit of 29.17 ± 0.16°C at 0 m water depth, obtained from local CTD data sets [24,27], was set for our study site. All equations, for the planktonic species, which yielded calculated temperatures above this value were excluded.
3. A lower temperature limit for the depth at the study site (i.e. 487 m) was set at 10.27 ± 0.17°C, obtained from multiple local CTD datasets [24,26,27]. All equations, for the benthic species which yielded calculated temperatures >1°C either side of this limit were excluded. This limit was arbitrarily set, as due to the steep slope of the CTD temperature profile at this depth a ±1°C restriction sets a depth boundary of ± 100 m.

Again, seasonal (summer and winter) CTD data was used to assign depths to the calculated temperatures with an overall average taken to define the Mg/Ca-ACD values.

## Results and discussion

### Geochemical data

**Fig 6:**
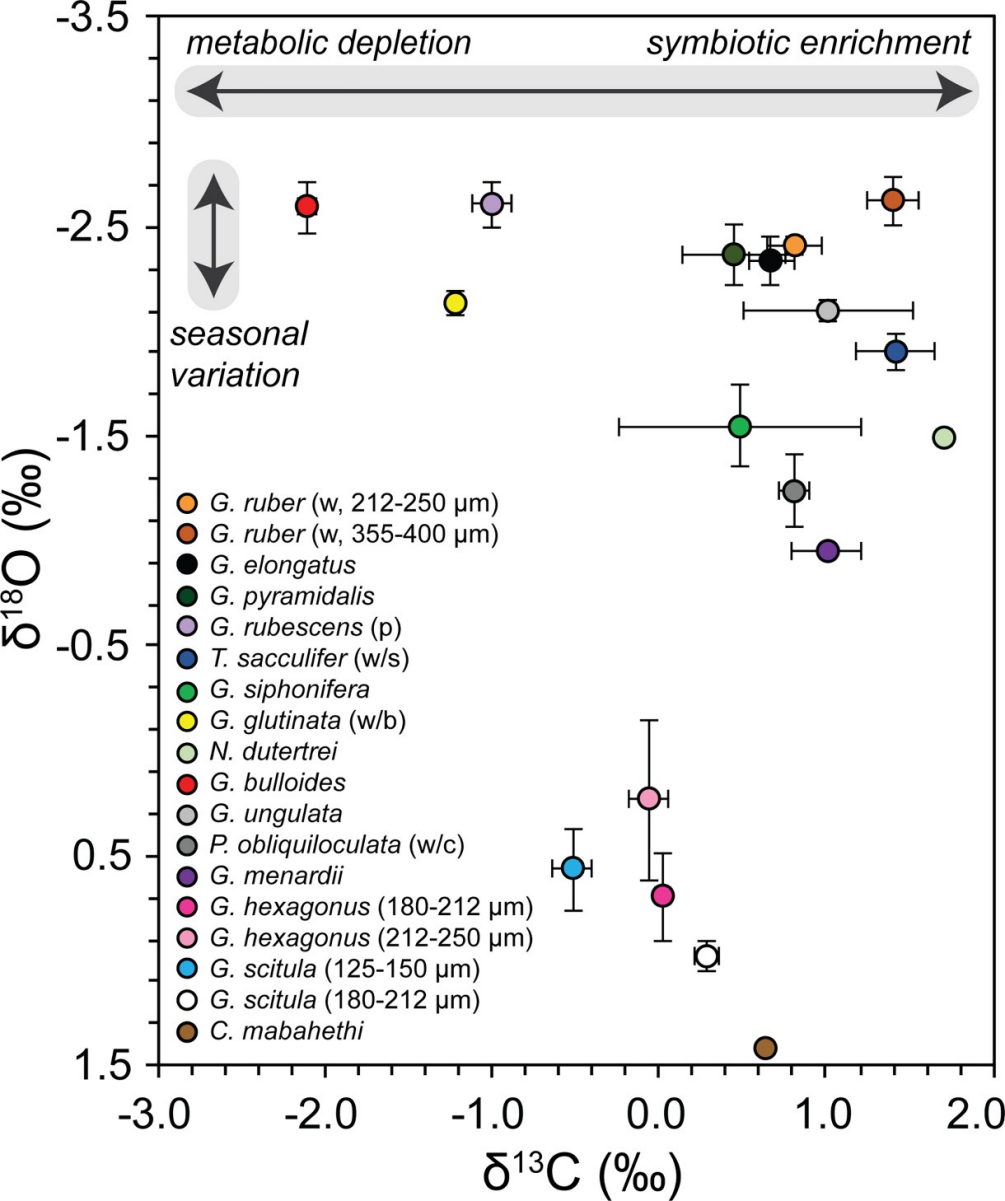
Mean δ^18^O and δ^13^C multi-species scatter plot with standard deviations (black bars) shown for all species. Interpretations in grey after [54]. w = white; p = pink; w/s = with sac; w/b = with bulla; w/c = with cortex.

The sedimentary record is an accumulation of foraminiferal tests, which can be from different seasons and represent different stages of their ontogeny as foraminifera migrate through the water column throughout their life cycles recording different geochemical signatures [1]. Thus, as anticipated, replicates of all species show some variability for both δ^18^O_c_ and δ^13^C_c_. The mean isotopic values with standard deviations (SD) illustrated in Fig 6, show the 14 planktonic species roughly plot in the expected (as per reports in the literature, e.g. [12,16,17]) vertical order with respect to their δ^18^O_c_ signatures (Table 3). The δ^13^C values of three surface dwelling species (*G*. *glutinata* (w/b), *G*. *bulloides* and *G*. *rubescens* (p)) are depleted, whereas symbiont enrichment is evident in the five symbiont-bearing species (*G*. *ruber* (w), *G*. *pyramidalis*, *G*. *elongatus*, *T*. *sacculifer* (w/s) and *G*. *siphonifera*). The δ^18^O_c_ signatures of the larger *G*. *ruber* (w) specimens (355–400 μm) are marginally lower than the smaller specimens (Δ = -0.22 ‰), yet the δ^13^C_c_ values are higher for the former (Δ = 0.58 ‰). The smaller *G*. *scitula* specimens (125–150 μm) have a lower δ^18^O_c_ value, and thus are interpreted to sit shallower in the water column compared with the pre-adult size (180–212 μm) with a more positive value (Δ = 0.42 ‰). A similar disparity (Δ = 0.80 ‰) is noted for their δ^13^C_c_ values. On the contrary, the small and large specimens of *G*. *hexagonus* display near identical δ^13^C values, yet a large range in δ^18^O_c_ values occurs with overlap of the signatures of the smaller and larger specimens. The benthic species, *C*. *mabahethi*, has the highest δ^18^O_c_ and lowest Mg/Ca values of 1.43 ‰ and 3.28 mmol/mol, respectively. Whereas, the symbiont bearing *G*. *ruber* (w, 355–400 μm) and non-symbiont bearing *G*. *bulloides* have the most depleted δ^18^O_c_ signature of -2.63±0.12 ‰ and highest Mg/Ca ratio of 7.41 mmol/mol, respectively.

**Table 3:** Raw δ^18^O, δ^13^C and Mg/Ca values for the 15 investigated species.

Species	Stable Isotopes (‰)						Mg/Ca (mmol/mol)	
	R1		R2		R3		R1	R2
	δ^13^C	δ^18^O	δ^13^C	δ^18^O	δ^13^C	δ^18^O		
*G*. *ruber* (w)^1^	0.92	-2.47	0.94	-2.40	0.57	-2.35	5.66	5.65
*G*. *ruber* (w)^2^	1.60	-2.66	1.25	-2.75	1.33	-2.47	-	-
*G*. *elongatus*	0.86	-2.40	0.61	-2.17	0.53	-2.43	-	-
*G*. *pyramidalis*	0.01	-2.55	0.60	-2.38	0.72	-2.19	-	-
*G*. *rubescens* (p)	-1.16	-2.72	-0.88	-2.64	-0.95	-2.47	-	-
*T*. *sacculifer* (w/s)	1.74	-2.03	1.17	-1.87	1.36	-1.83	5.09	4.55
*G*. *siphonifera*	-0.16	-1.36	0.11	-1.81	1.50	-1.48	5.49	-
*G*. *glutinata* (w/b)	-1.25	-2.20	-1.21	-2.08	-	-	-	-
*N*. *dutertrei*	1.67	-1.49	1.75	-1.48	-	-	-	-
*G*. *bulloides*	-2.14	-2.45	-2.16	-2.75	-2.04	-2.58	7.41	-
*G*. *ungulata*	1.30	-2.16	1.42	-2.08	0.28	-2.05	-	-
*P*. *obliquiloculata* (w/c)	0.84	-1.36	0.89	-1.00	0.70	-1.38	4.31	4.41
*G*. *menardii*	1.21	-0.96	0.80	-0.95	-	-	4.45	2.94
*G*. *hexagonus*^3^	0.08	0.58	-0.02	0.52	-0.01	0.98	-	-
*G*. *hexagonus*^1^	-0.12	0.05	0.10	0.78	-0.17	-0.11	-	-
*G*. *scitula*^4^	-0.64	0.37	-0.41	0.76	-	-	-	-
*G*. *scitula*^3^	0.36	0.91	0.21	1.05	-	-	-	-
*C*. *mabahethi/wuellerstorfi*	0.65	1.43	-	-	-	-	3.28	-

w = white; p = pink; w/s = with sac; w/b = with bulla; w/c = with cortex

1: 212–250 μm

2: 355–400 μm

3: 180–212 μm

4: 125–150 μm.

The letter ‘R’ denotes replicate measurements.

### Seawater δ^18^O

**Fig 7:**
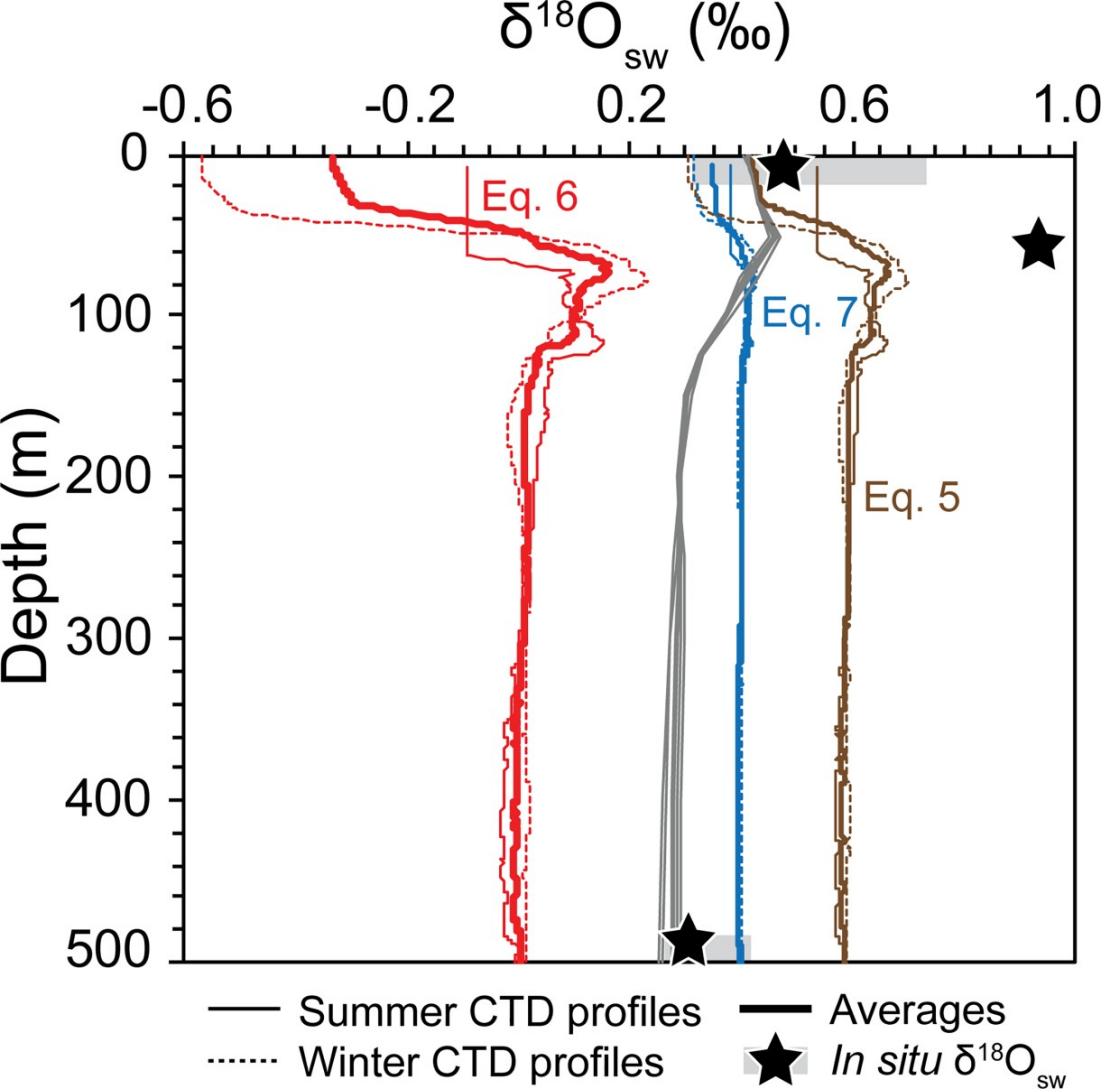
Calculated vertical δ^18^O_sw_ depth profiles derived using the salinity Eqs 5, 6 and 7 of [49], [47] and [50], respectively. Summer (thin solid lines) and winter (thin dashed lines) salinity profiles of [26] and [27], respectively were used to show the seasonality and calculate overall averages (thick solid lines). The gridded δ^18^O_sw_ data set by [51] for the Maldives region is shown for reference in grey. Black stars show mean measured δ^18^O_sw_ values from the region for the surface (0 m) by [45–47], at 50 m by [55] and at ±500 m by [48] with the range in values represented by the grey shaded boxes. Equation numbers are identified on the graph in their respective colors.

The salinity Eqs 5, 6 and 7 of [49], [47] and [50] all produce vertical seasonal δ^18^O_sw_ profiles, which visually look identical, yet the absolute values are significantly offset (Fig 7). Measured *in situ* regional δ^18^O_sw_ values for the surface (0 m) by [45–47], at 50 m by [55] and at ±500 m by [48] were used to select the most applicable equation for the study site.

Eq 5 of [49] calculates summer and winter profiles with positive surface values with an average of 0.42 ‰ which is comparable to the *in situ* measurements. On the contrary the bottom water δ^18^O_sw_ is over estimated (average at 500 m = 0.59 ‰) and is out of the local range measured by [48], (Fig 7). The Eq 6 of [47] produces summer and winter profiles with negative values for the top 50 m of the water column with average values of -0.33 ‰ and 0.01 ‰ at the surface and 500 m water depth, respectively. Overall, the profiles have significantly lower values in comparison to the *in situ* data. On the contrary, all calculated values are positive using the Eq 7 of [50] with average values of 0.35 ‰ and 0.40 ‰ at the surface and 500 m water depth, respectively. The latter equation calculates δ^18^O_sw_ values comparable to the measured regional surface δ^18^O_sw_ data of [45–47], with a range of δ^18^O_sw_ from 0.32 to 0.74 ‰, and an average δ^18^O_sw_ = 0.49 ‰. They were also comparable to the regional δ^18^O_sw_ values measured at ±500 m water depth by [48] with a range of δ^18^O_sw_ from 0.26 to 0.42 ‰, and an average δ^18^O_sw_ = 0.30 ‰.

The calculated δ^18^O_sw_ profiles are also compared with the gridded dataset of [51] from the Maldives region (Fig 7). The Eq 7 of [50] derived values are most similar with the gridded dataset. These calculated profiles have slightly lower values than the gridded dataset for the top 80 m and marginally higher values down to 500 m. There is, however, a lack of measured subsurface δ^18^O_sw_ data from the northern Indian Ocean. The low spatial and depth resolution of the subsurface data significantly limits the interpretation and application of the gridded dataset and thus we used this dataset solely to verify the calculated profiles.

Overall, all equations underestimate the δ^18^O_sw_ at 50 m with none of the calculated profiles having values corresponding to the measured value by [55] at 50 m. Nevertheless, considering its correlation with the *in situ* surface and bottom water δ^18^O_sw_, the salinity Eq 7 of [50] is deemed most suitable for this study. The δ^18^O_sw_ average for the SML down to a depth of 75 m, coinciding with the maximum salinities, is 0.38 ‰ with an average of 0.40 ‰ for 75–500 m water depth. The winter and summer averages for the same ranges are 0.36 ‰, 0.40 ‰ and 0.39 ‰, 0.40 ‰, respectively.

### Method one: Isotope-ACD estimates

As stated above, Method one tests the applicability of using individual species-specific δ^18^O-temperature equations (e.g. [45], [56], [57], [15], [58]) versus applying Eqs 1–4 for all species as well as different δ^18^O_sw_ values in two separate applications: Method 1.1 and 1.2.

### Method 1.1: δ^18^O-temperature and isotope-ACD allocations

In Method 1.1 when using a single δ^18^O_sw_ value of 0.39 ‰ (Table 4), allocated based on an average of published values for 0 m and ±500 m from the Maldives region [45–48], Eqs 1, 3 and 4 calculate high temperatures and thus result in shallower ACD allocations for all assumed shallow-dwelling species (Table 1) in comparison to Eq 2. Additionally, calculated temperatures, for *G*. *ruber* (w, 355–400 μm), *G*. *rubescens* (p) and *G*. *bulloides* are above the local CTD measurements for Eqs 1 and 4 and as such have no assigned ACDs. When assessing the first four species (*G*. *ruber* (w), *T*. *sacculifer*, *N*. *dutertrei* and *G*. *bulloides*) with calibrated species-specific equations ([45], [56], [57]), Eqs 1, 3 and 4 derived ACDs are considerably shallower than the species-specific and Eq 2 ACD allocations. The largest differences are noted for *G*. *ruber* (355–400 μm) and *G*. *bulloides*.

**Table 4:** Temperature calculations and seasonally averaged ACD estimates using different δ^18^Oc–temperature equations and an average δ^18^O_sw_ value for all species (Method 1.1).

Species	δ^18^O_sw_ (VSMOW)	Equation Reference									
		^a^Eq 1		^b^Eq 2		^c^Eq 3		^d^Eq 4		Species-specific Eqs.	
		T (°C)	ACD (m)	T (°C)	ACD (m)	T (°C)	ACD (m)	T (°C)	ACD (m)	T (°C)	ACD (m)
*G*. *ruber* (w)^1^	0.39	28.84	62±2	25.59	77±4	28.41	67±2	28.96	51±16	^e^25.65	76±4
*G*. *ruber* (w)^2^	0.39	29.85	N/A	26.60	74±2	29.52	22	30.03	N/A	^e^ 26.73	74±2
*G*. *elongatus*	0.39	28.50	66±2	25.25	78±4	28.04	69±1	28.60	66±2		
*G*. *pyramidalis*	0.39	28.66	65±2	25.41	77±4	28.21	68±2	28.77	63±2		
*G*. *rubescens* (p)	0.39	29.77	N/A	26.52	74±2	29.43	55±6	29.94	N/A		
*T*. *sacculifer* (w/s)	0.39	26.52	74±2	23.29	83±7	25.88	76±4	26.53	74±2	^e^ 22.88	84±7
*G*. *siphonifera*	0.39	24.85	80±4	21.66	92±4	24.10	81±6	24.82	80±4		
*G*. *glutinata* (w/b)	0.39	27.58	70±1	24.34	81±5	27.03	72±1	27.64	70±0		
*N*. *dutertrei*	0.39	24.55	81±5	21.37	97±4	23.77	82±6	24.51	81±5	^f^21.38	97±4
*G*. *bulloides*	0.39	29.69	N/A	26.44	74±3	29.34	53±10	29.86	N/A	^g^27.24	72±1
*G*. *ungulata*	0.39	27.40	71±0	24.16	81±6	26.84	73±2	27.45	71±0		
*P*. *obliquiloculata* (w/c)	0.39	23.45	83±7	20.30	102±6	22.61	84±7	23.40	83±7	^h^23.93	82±6
*G*. *menardii*	0.39	22.11	88±7	19.00	113±6	21.18	98±6	22.04	89±6	^f^20.25	104±8
*G*. *hexagonus*^3^	0.39	14.63	183±20	11.88	306±29	13.46	223±23	14.71	182±20		
*G*. *hexagonus*^1^	0.39	16.69	139±6	13.82	208±26	15.56	166±15	16.69	139±6		
*G*. *scitula*^4^	0.39	15.22	172±13	12.43	271±31	14.05	199±24	15.27	170±14		
*G*. *scitula*^3^	0.39	13.35	228±22	10.69	420±32	12.18	285±27	13.50	221±23		
*C*. *mabahethi*	0.39	11.35	344±24	8.84	755	10.18	497±16	11.63	321±27	^i^9.86	533±10

^a^The *T*. *sacculifer* Eq 1 of [37]

^b^The multi-species Eq 2 of [38]

^c^The inorganic calcite Eq 3 of [39]

^d^The synthetic calcite Eq 4 of [40]

^e^ -specific Eqs. of [45]

^f^ -specific Eqs. of [56]

^g^ -specific Eqs. of [57]

^h^ -specific Eqs. of [15]

^i^ -specific Eqs. of [58].

w = white; p = pink; w/s = with sac; w/b = with bulla; w/c = with cortex

1: 212–250 μm

2: 355–400 μm

3: 180–212 μm

4: 125–150 μm.

The average δ^18^O_sw_ was calculated based on measured δ^18^O_sw_ values from the northern Indian Ocean from 0 m and ±500 m [45–48]. ACDs were assigned using CTD data from the Maldives region for summer [24,26] and winter [27]. Grey shading denotes the equations identified as most suitable for the ACD calculations.

On the contrary, the species-specific derived ACDs for the intermediate dwellers *P*. *obliquiloculata* and *G*. *menardii* are not as cohesive with Eq 2. The assigned ACD, using the species-specific equation of [15], for the former species is more comparable to the inferred ACDs from Eqs 1, 3 and 4 than to Eq 2. However, as the *P*. *obliquiloculata* species-specific equation from [15] is based on modelled data which shows a large spread and relatively low correlation, we still consider Eq 2 as most suitable for use in accordance with the other shallow to intermediate-dwellers. Furthermore, both Eqs 2 and 3 result in assigned ACDs comparable to the species-specific allocation for *G*. *menardii*, however, similarly to above, we still consider the former most applicable for use with this intermediate-dwelling species.

The benthic allocation from Eq 3 (497±16 m) is directly comparable to the study site depth of 487 m. It is apparent though that the allocated δ^18^O_sw_ is possibly marginally too low for use with the deeper-dwelling sub-thermocline species, as the species-specific benthic equation of [58] produced a deeper ACD estimate (533±10 m) than the study site. On the contrary, Eqs 1 and 4 benthic ACD allocations are significantly shallower, with the Eq 2 estimates exceedingly deep and as such these Eqs. do not appear suitable for application with the deeper-dwelling sub-thermocline species. In this instance, however, a higher δ^18^O_sw_ value could offset the use of an equation calibrated for a SML species.

Calculations were then repeated for Method 1.1, using the calculated δ^18^O_sw_ values obtained using the salinity Eq 7 of [50] (Table 5). A δ^18^O_sw_ value of 0.38 ‰, averaged for the top 75 m water depth, is used for the reported shallow (SML) and intermediate- (thermocline) dwelling species. Whereas, a δ^18^O_sw_ value of 0.40 ‰, averaged for 75–500 m water depth, is used for the reported deeper-dwelling (thermocline to sub-thermocline) species. The delineation at 75 m was assigned based on the salinity maxima from local CTD data (Fig 3; [26,27]).

**Table 5:** Temperature calculations and seasonally averaged ACD estimates using different δ^18^O_c_−temperature equations and different δ^18^O_sw_ values for the reported shallow-intermediate and deeper-dwelling species (Method 1.1).

Species	δ^18^O_sw_ (VSMOW)	Equation Reference									
		^a^Eq 1		^b^Eq 2		^c^Eq 3		^d^Eq 4		Species-specific Eqs.	
		T (°C)	ACD (m)	T (°C)	ACD (m)	T (°C)	ACD (m)	T (°C)	ACD (m)	T (°C)	ACD (m)
*G*. *ruber* (w)^1^	0.38	28.80	44±27	25.55	77±4	28.36	67±2	28.91	42±29	^e^25.60	77±4
*G*. *ruber* (w)^2^	0.38	29.81	N/A	26.55	74±2	29.47	37	29.98	N/A	^e^26.68	74±2
*G*. *elongatus*	0.38	28.45	67±2	25.20	78±4	27.98	69±1	28.55	66±2		
*G*. *pyramidalis*	0.38	28.61	66±2	25.37	77±4	28.16	68±1	28.72	64±1		
*G*. *rubescens* (p)	0.38	29.73	N/A	26.47	74±2	29.38	57±5	29.89	N/A		
*T*. *sacculifer* (w/s)	0.38	26.47	74±2	23.25	83±7	25.83	76±4	26.49	74±2	^e^22.83	84±7
*G*. *siphonifera*	0.38	24.81	80±4	21.62	93±3	24.05	81±6	24.77	78±3		
*G*. *glutinata* (w/b)	0.38	27.53	71±1	24.29	81±5	26.98	73±1	27.59	70		
*N*. *dutertrei*	0.38	24.50	81±5	21.32	98±4	23.72	82±6	24.47	81±5	^f^21.32	98±4
*G*. *bulloides*	0.38	29.65	N/A	26.39	74±3	29.29	52±11	29.81	N/A	^g^27.19	72±1
*G*. *ungulata*	0.38	27.35	71±1	24.12	81±6	26.79	73±1	27.40	71		
*P*. *obliquiloculata* (w/c)	0.38	23.41	83±7	20.25	104±8	22.56	85±7	23.35	83±7	^h^23.88	82±6
*G*. *menardii*	0.38	22.07	88±7	18.95	116±4	21.14	99±5	21.99	89±7	^f^20.20	104±7
*G*. *hexagonus*^3^	0.40	14.67	183±20	11.92	303±30	13.50	221±23	14.75	182±19		
*G*. *hexagonus*^1^	0.40	16.74	139±6	13.86	207±26	15.60	165±15	16.74	139±6		
*G*. *scitula*^4^	0.40	15.26	170±14	12.47	270±31	14.10	198±24	15.32	169±14		
*G*. *scitula*^3^	0.40	13.40	225±23	10.73	414±36	12.22	281±30	13.54	220±23		
*C*. *mabahethi*	0.40	11.40	339±26	8.88	754	10.23	496±10	11.67	318±27	^i^9.91	527±5

^a^The *T*. *sacculifer* Eq 1 of [37]

^b^The multi-species Eq 2 of [38]

^c^The inorganic calcite Eq 3 of [39]

^d^The synthetic calcite Eq 4 of [40]

^e^ Species-specific Eqs. of [45]

^f^ -specific Eqs. of [56]

^g^ -specific Eqs. of [57]

^h^ -specific Eqs. of [15]

^i^ -specific Eqs. of [58].

w = white; p = pink; w/s = with sac; w/b = with bulla; w/c = with cortex

1: 212–250 μm

2: 355–400 μm

3: 180–212 μm

4: 125–150 μm.

The δ^18^O_sw_ estimates were averaged from the calculated δ^18^O_sw_ profile using the salinity Eq 7 of [50]. ACDs were assigned using CTD data from the Maldives region for summer [24,26] and winter [27]. Grey shading denotes the equations identified as most suitable for the ACD calculations.

The allocation of marginally different δ^18^O_sw_ values for the SML and deeper depths results in slight differences in temperature calculations and subsequent ACD assignments. As the δ^18^O_sw_ value for the surface and sub-surface depths is similar to the first calculations, minimal differences in ACDs are noted for these shallow to intermediate-dwelling species. Eq 2 derived ACDs are still most comparable with those allocated using the species-specific equations, with again the exception of *P*. *obliquiloculata*. Additionally, ACDs allocated using Eqs 1, 3 and 4 have estimates which are again significantly shallower. On the contrary, as noted above, using a higher δ^18^O_sw_ value for the thermocline and sub-thermocline species results in shallower benthic ACD estimates. In particular, the benthic ACD estimate when using Eq 3 (496±10 m) is again most comparable to the study site with the species-specific equation providing a slightly deeper ACD estimate of 527±5 m. We use the equation of [58], calibrated for the genera *Cibicidoides* and *Planulina*, for our benthic species, *C*. *mabahethi*. It is assumed that both the *Cibicidoides* and *Planulina* genera calcify in near equilibrium to the surrounding seawater. It is, however, questionable whether all benthic species precipitate in isotopic equilibrium with the seawater. Thus, when applying the equation of [58], small differences between the *Cibicidoides* and *Planulina* genera and the investigated species, *C*. *mabahethi* could account for these deeper ACD estimates. Furthermore, our utilized δ^18^O_sw_ value of 0.40‰ could still be too low, especially considering [48] measured a range in δ^18^O_sw_ values (0.26–0.42 ‰) within the Maldives Inner Sea.

Therefore, we conclude that, if using a single δ^18^O_sw_ value or an averaged value for the SML and thermocline to sub-thermocline waters, it is plausible to use Eq 2 of [38] for surmised shallow to intermediate-dwelling species and Eq 3 of [39] for all other deeper-dwelling (thermocline to sub-thermocline) species ACD calculations as applied in [17]. Applying only species-specific equations would still be the preferred method yet, as they are not always available, their use to crosscheck and validate a chosen single equation to utilize for all species is advised.

It should be noted that care must be taken when allocating the δ^18^O_sw_ values for use in the δ^18^O-temperature equations. In this particular instance if only *in situ* shallow water (0–50 m) measurements are taken into consideration, averages of 0.58–0.70 ‰ would be obtained. The deeper water salinity maximum would skew the data and using a considerably higher δ^18^O_sw_ allocation, in the calculations, would result in significantly different ACD estimates. In this instance, we took an average from the surface (0 m) and bottom (±500 m) water measurements, which avoids unintentionally over estimating the δ^18^O_sw_ value.

### Method 1.2: δ^18^O_e_ profiles and isotope-ACD allocations

**Fig 8:**
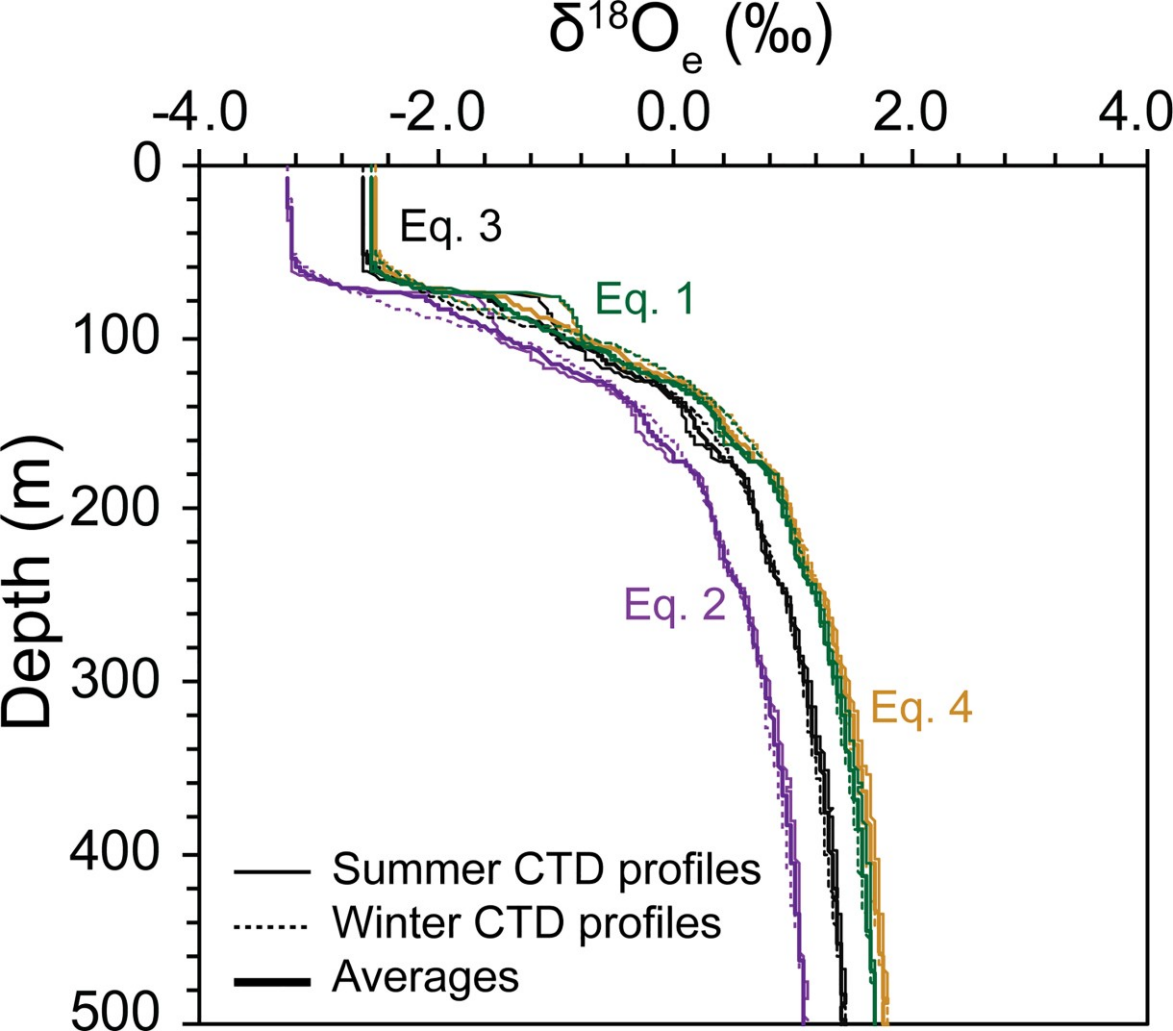
Calculated vertical δ^18^O_e_ depth profiles derived using δ^18^O-temperature Eqs 1, 2, 3 and 4 of [37], [38], [39] and [40], respectively. All profiles were calculated using the δ^18^O_sw_ profiles obtained from Eq 7 shown in Fig 7. Seasonal variations are represented by the thin lines (summer: solid lines; winter: dashed lines) with the thicker lines representing the overall averages. Equation numbers are identified on the graph in their respective colors.

For Method 1.2, seasonal δ^18^O_sw_ profiles, calculated using the salinity Eq 7 of [50], are used together with Eqs 1–4 in order to generate vertical δ^18^O_e_ profiles (Fig 8). Eq 2 calculates the lowest δ^18^O_e_ values for the top ±80 m with the surface waters being 0.62–0.72 ‰ lower than the values derived when using Eqs 1, 3 and 4. The latter three δ^18^O_e_ profiles are comparable down to ~120 m, after which the Eq 3 derived graph is consistently lower and Eq 4 has the highest values, yet only marginally in comparison to Eq 1.

ACDs are subsequently allocated using the seasonal vertical δ^18^O_e_ profiles produced in Fig 8. In addition, as conducted by [14] selected species-specific equations are used to further assess the applicability of the other four equations (Table 6). As expected, when using the Eqs 1, 3 and 4 derived graphs, the shallow to intermediate-dwelling species have the shallowest ACDs, which are consistently shallower in comparison with the species-specific allocations. On the contrary, ACDs assigned using the Eq 2 derived graph, are nearly identical with the allocated ACD values for the four shallow-dwelling species with species-specific equations (*G*. *ruber*, *T*. *sacculifer*, *N*. *dutertrei and G*. *bulloides*). Species-specific equations are available for two deeper, thermocline species (*P*. *obliquiloculata* and *G*. *menardii*) and for the former the derived ACDs are on the contrary more comparable with those derived from Eqs 1, 3 and 4. Yet, similarly to above, this species is anomalous as the species-specific equation was obtained from modelled data and as such, we chose to allocate Eq 2 as most applicable.

**Table 6:** Seasonally averaged isotope-ACDs assigned using δ^18^O_e_ curves calculated using different δ^18^O_c_−temperature Eqs 1–4 and species-specific Eqs. in conjunction with the generated δ^18^O_sw_ curves from Eq 7 [50], (Method 1.2).

Species	Equation References for Allocated ACDs (m)				
	^a^Eq 1	^b^Eq 2	^c^Eq 3	^d^Eq 4	Species-specific Eqs.
*G*. *ruber* (w)^1^	62±3	78±4	66±2	61±3	^e^78±4
*G*. *ruber* (w)^2^	N/A	74±1	13±13	N/A	^e^73±1
*G*. *elongatus*	65±2	78±4	67±2	64±3	
*G*. *pyramidalis*	64±2	78±4	66±2	63±3	
*G*. *rubescens* (p)	N/A	75±2	54±7	N/A	
*T*. *sacculifer* (w/s)	75±2	83±8	77±3	75±2	^e^83±9
*G*. *siphonifera*	79±5	91±5	81±6	80±5	
*G*. *glutinata* (w/b)	70	81±6	73±1	70	
*N*. *dutertrei*	80±5	97±1	82±7	81±6	^f^95±2
*G*. *bulloides*	N/A	75±2	57±6	N/A	^g^71±1
*G*. *ungulata*	71	81±6	73±1	71	
*P*. *obliquiloculata* (w/c)	82±7	105±2	84±9	82±7	^h^83±8
*G*. *menardii*	86±9	117±4	101±2	87±8	^f^106±2
*G*. *hexagonus*^3^	169±4	283±4	205±1	168±4	
*G*. *hexagonus*^1^	135±2	188±3	156±7	135±2	
*G*. *scitula*^4^	161±6	246±1	180±2	160±6	
*G*. *scitula*^3^	209±2	392±25	260±5	201	
*C*. *mabahethi*	323±14	755	479±11	300±7	^i^528±5

^a^The *T*. *sacculifer* Eq 1 of [37]

^b^The multi-species Eq 2 of [38]

^c^The inorganic calcite Eq 3 of [39]

^d^The synthetic calcite Eq 4 of [40]

^e^ Species-specific Eqs. of [45]

^f^ Species-specific Eqs. of [56]

^g^ Species-specific Eqs. of [57]

^h^ Species-specific Eqs. of [15]

^i^ Species-specific Eqs. of [58].

w = white; p = pink; w/s = with sac; w/b = with bulla; w/c = with cortex

1: 212–250 μm

2: 355–400 μm

3: 180–212 μm

4: 125–150 μm.

Grey shading denotes the equations selected as most suitable for the ACD calculations.

Finally, the benthic ACD allocations vary significantly when comparing all five estimates. As the study site depth is shallow, and known at 487 m, it is apparent that the benthic estimate using Eq 3 is most comparable. ACDs derived using Eqs 1 and 4 are considerably shallower whereas a significantly deeper ACD is assigned when using the δ^18^O_e_ profile from Eq 2. The *Cibicidoides and Planulina* equation of [58] estimates an ACD of 528±5 m, which again is deeper than the known depth of the study site.

Similarly, to Method 1.1, the best approach to derive δ^18^O_e_ values is to use species-specific equations. However, as these are not always available for all investigated species a single equation is required for use with multiple species. Overall, we conclude that Eqs 2 and 3 of [38] and [39] are most applicable for our dataset, as previously mentioned when using Eq 1 of [37] and Eq 4 of [40] the assigned ACDs are all consistently too shallow. Thus, based on their agreement with the species-specific derived ACDs and benthic allocations, and similarly to Method 1.1, we found it suitable to use Eq 2 for the shallow to intermediate-dwelling (SML-thermocline) species and Eq 3 for the deeper-dwelling (thermocline to sub-thermocline) species. Alternatively, Eq 3 could be applied for all species yet there is the possibility of slightly shallower ACD estimates for the SML dwelling species.

### Method two: Mg/Ca-ACD estimates

**Fig 9:**
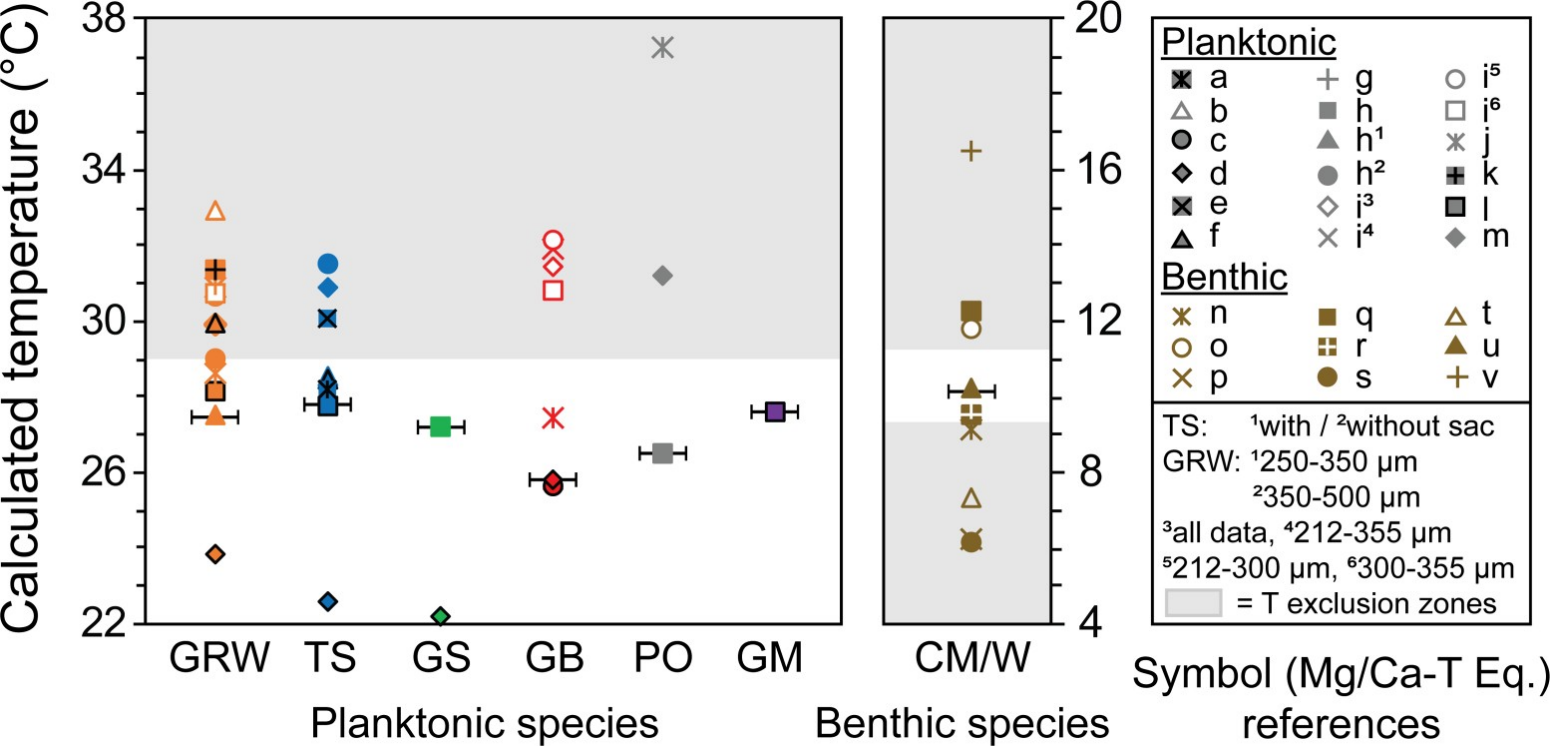
Range in temperature calculations using various species-specific Mg/Ca-temperature equations for six planktonic and one benthic species. GRW: *G*. *ruber* (w), TS: *T*. *sacculifer* (w/s), GS: *G*. *siphonifera*, GB: *G*. *bulloides*, PO: *P*. *obliquiloculata* (w/c), GM: *G*. *menardii* & CM/W: *C*. *mabahethi/wuellerstorfi* with w = white, w/s = with sac, w/c = with cortex, grey shading indicates temperature (T) exclusion zones as outlined in the methods section. Horizontal black lines indicate the selected equation for each species. See Table 7 for species-specific equation references which are also indicated by a * below. (Planktonic references a: [59], b: [60]*, c: [61], d: [62], e: [63], f: [64], g: [65], h: [13]*, i: [66], j: [67], k: [68], l: [69]*, m: [70]; Benthic references n: [71], o: [52], p: [72], q: [73], r: [74], s: [53], t: [75], u: [76]*, v: [77]. See S2 Table for more information regarding each equation.

There is a large spread in the temperature estimates from the applied species-specific equations (Fig 9). As previously mentioned, this was anticipated due to the large variability in the calibration parameters (e.g. geographical location, temperature calibration range, specimen cleaning method) and the quadratic nature of the Mg/Ca-temperature equations. The choice of applicable equation is significant as the subsequent ACDs can be quite offset. Suitable species-specific Mg/Ca-temperature equations were, therefore, firstly constrained using the steps outlined in the Methods section above (Fig 9). Following this, all factors were assessed to select the species-specific equations most suitable for our study and study site (Fig 9). The subsequent Mg/Ca-ACD allocations are somewhat comparable to the isotope-ACDs (Table 7). Some disparity was anticipated between the two methods, as the isotopic data incorporates a salinity effect and were additionally not corrected for species-specific isotopic offsets.

**Table 7:** Assigned, seasonally averaged Mg/Ca-ACDs (Method 2).

Species	Reference	ACDs (m)
*G*. *ruber* (w, 212–250 μm)	[13]*	70 ± 1
*T*. *sacculifer* (w/s)	[69]	70
*G*. *siphonifera*	[13]	72 ± 1
*G*. *bulloides*	[60]	76 ± 4
*P*. *obliquiloculata* (w/c)	[13]	74 ± 3
*G*. *menardii* (R1)	[69]	70 ± 1
*G*. *menardii* (R2)	[69]	83 ± 7
*C*. *mabahethi/wuellerstorfi*	[76]	494 ± 17

*calibrated for the 250–350 μm size fraction, w = white, w/s = with sac, w/c = with cortex

ACDs were allocated using CTD data from the Maldives region for summer [24,26] and winter [27]. R = replicate, w = white, w/s = with sac, w/c = with cortex.

The Mg/Ca-ACDs for all species, except *G*. *bulloides*, are shallower than their isotope-ACD counterparts. This could be attributable to the natural geochemical variability incurred through the measurement of different specimens. Additionally, a possible salinity influence for the surface dwellers *G*. *ruber* (w), *T*. *sacculifer* (w/s) and *G*. *siphonifera* could account for the marginal Mg/Ca- and isotope-ACD differences ranging between 7–20 m. *Globigerina bulloides* has comparable Mg/Ca- and isotope-ACD estimates. Yet for the deeper dwellers, *P*. *obliquiloculata* and *G*. *menardii* the isotope- and Mg-/Ca-ACDs vary significantly. In this instance, we attribute the differences not to hydrological conditions but instead to the applied equations and species morphology. Particularly for these two species, there are few species-specific calibrated equations and as such, we are limited in the choice of equations to apply. Furthermore, both species precipitate secondary calcite, for *G*. *menardii* as an encrusted keel and for *P*. *obliquiloculata* in the form of a cortex. As such, using variable specimens for the respective geochemical analyses would result in further variability in the geochemistry and subsequently their inferred ACDs. This is particularly evident in the variation of the *G*. *menardii* replicates. As the measurements for this species were highly variable, in comparison to the other replicate datasets, we did not calculate an average but instead treated each replicate separately. The resultant ACDs range from 70–90 m, which, even though are still shallower than the isotope-ACD allocation, reflect the natural variation within the species.

We further attribute the difference in isotope-ACD and Mg/Ca-ACD allocations for *P*. *obliquiloculata* to the differential Mg/Ca distributions between the species inner test and outer cortex layer. Using Laser–ablation inductively coupled plasma–mass spectrometry (LA-ICP-MS), [78] showed the cortex Mg/Ca values are around 3–10 times lower in comparison to the inner test geochemistry. Furthermore, the cortex thickness was shown to be variable and as such, its influence on bulk geochemistry measurements would vary. Therefore, as a consequence of using different species for the isotopic and geochemical measurements in this study, in conjunction with having to pool multiple specimens for the various measurements could account for these different ACD allocations for *P*. *obliquiloculata*.

### Planktonic foraminifera ACD allocations

**Fig 10:**
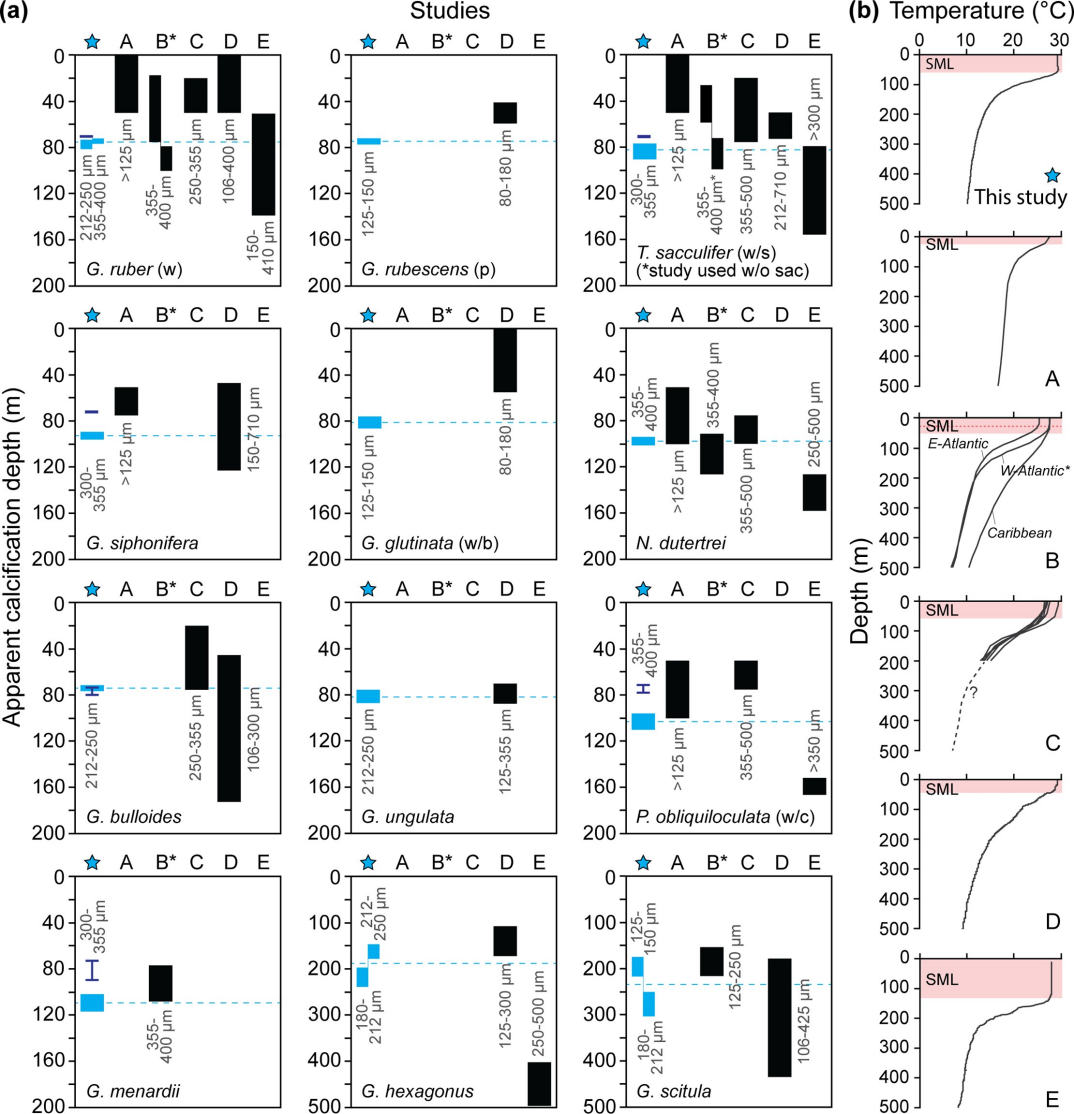
**(a) Comparison of the ACDs calculated from this study (blue star: blue blocks denote the range in isotope-ACDs averaged from the best options from both Methods 1.1 and 1.2 and dark blue bars show the Mg/Ca-ACDs obtained from Method 2) and the five (sub)tropical global studies (black blocks) with (b) showing the vertical thermal structure through the water column at each study location.** Grey text denotes the size of the tests used for each study. The blue dashed lines indicate the average isotope-ACDs for this study. Data sources: A [13]; B [16]; C [14]; D [12] and E [17] with only the west Atlantic (W-Atlantic) derived ACDs shown for study B. SML = surface mixed layer, w = white, p = pink, w/s = with sac, w/o = without, w/b = with bulla, w/c = with cortex.

Regionally distinct ecological niches develop due to differences in water temperature, nutrients, food availability, predation and light intensity all of which contribute to the vertical dispersion of planktonic foraminifera. Understanding these distributions is thus important for regional paleoceanographic reconstructions (e.g. [2,17]). Important to consider is planktonic foraminifera living depths are not globally ubiquitous [2,3]. This can be due to differing quality and quantity of available prey, different genotypes [79] or hydrographic variability. Additionally, ACD calculation methods and sampling strategies differ across authors, such as sampling at different times during species ontogenic cycles or targeting different species-specific size ranges. These can all incorporate further discrepancies into the foraminifera ACD estimates that are demonstrated in the comparison of Fig 10.

The five global, low latitude ACD studies referenced in this work (i.e. studies A-E [12–15,17], Fig 1), were selected as they have different regional hydrological controls, and used different methods and equations to calculate their foraminiferal ACD estimates. In addition, 12 common species were analysed yet were from variable size fractions reflecting different instances during each species ontogenetic cycles (Fig 10). It is, therefore, difficult to disentangle all contributions accounting for these observed ACD differences, however, as this study tested all authors’ methods and equations, some comment can still be made.

The first four studies [12–14,16] calculated isotope-ACDs, each utilizing a different combination of equations and δ^18^O_sw_ values. The largest deviations are noted for the surface-dwelling species, *G*. *ruber* (w), *G*. *rubescens* (p), *T*. *sacculifer* (w/s) and *G*. *glutinata* (w/b) which can in part be attributed to equation selection, thermocline depth differences and specimen selection (Fig 10). Studies A [13] and D [12] both have shallower SMLs (30–40 m). Additionally, they utilized Eqs 1 and 4 for all ACD calculations, which we found produced considerably shallower ACDs. Therefore, equation choice in conjunction with more condensed SMLs could account for the observed deviations. Additionally, we exclusively used *T*. *sacculifer* which displayed gametogenic features (i.e. a sac like final chamber) and *G*. *glutinata* specimens with bulla, which were recognized by [29,80,81] as possible reproductive structures. These species are known to migrate to deeper waters towards the end of their ontogenetic cycle, which is reflected in this study with comparably deeper ACDs for these two species.

On the contrary, in comparison with the present study, both studies B [16] and C [14] have comparable SML depths. The latter applied species-specific equations, however, used relatively large size ranges for each species, which could contribute to the large range and differences in the shallow-dwelling species ACDs. Study B of [16] calculated foraminifera ACDs for samples from three distinct regions in the Caribbean, east Atlantic Ocean and west Atlantic Ocean. Our thermocline structure is most similar to the latter region and thus for simplicity we only displayed these ACDs in the comparison of Fig 10. [16] used comparable size fractions, yet utilized Eq 4, which as expected results in marginally shallower ACDs estimates. For both *G*. *ruber* (w) and *T*. *sacculifer* calculations, they did report Eq 2 derived ACDs, which are accordingly slightly deeper and more comparable with our inferred depths (Fig 10). [2] reported deep shoaling (i.e. congregating) observations for some planktonic foraminifera in the subtropical eastern North Atlantic. This was attributed to subsequent deepening of the SML in summer (100–150 m) and association with increasing temperatures. The low latitudinal position of the Maldives, together with increased light and higher SST temperatures could support a deeper depth habitat of the surface dwellers in the tropical, Indian Ocean.

*Globigerinella siphonifera* and *G*. *ungulata* have comparable ACDs across the first four studies as do the typically thermocline and sub-thermocline dwelling *N*. *dutertrei*, *P*. *obliquiloculata* (w/c), *G*. *menardii*, *G*. *hexagonus* and *G*. *scitula* (Fig 10; [12–15]. The opportunistic species, *G*. *bulloides* shows a large spread (Fig 10); this is probably a combination of differences in calculation methods as well as a reflection of its wide-ranging depth habitat preferences as a result of differential seasonal upwelling conditions across the study locations.

The final isotope-ACD calculation method we selected was similar to that applied in study E of [17], albeit their final ACDs are derived from a combination of isotope- and Mg/Ca methods. In addition, they analysed specimens from a large size range, in contrast to the restricted size fractions used in this study. Both shallow-dwelling species, *G*. *ruber* (w) and *T*. *sacculifer* (w/s) have overlapping ACD ranges whereas deviations for *N*. *dutertrei*, *P*. *obliquiloquilata* (w/c) and *G*. *hexagonus* ACD allocations are apparent. Regional differences in thermocline and nutrient conditions can account for these differences. In the present study, the SML is shallower extending down to ~69 m water depth with a thermocline between ~70–120 m. On the contrary, [17] reported a SML extending down to 105 m and a thermocline between 130–230 m water depth in the West Pacific Warm Pool (WPWP) which could account for the deeper living depths for all species from study E.

### Northern equatorial Indian Ocean ACD controls

This study attempts to constrain foraminiferal ACDs for the northern equatorial Indian Ocean using a combination of stable isotopes and Mg/Ca ratios (explained in the sections above). Previous studies (e.g. [1,3]) have reported that the vertical peak in planktonic foraminifera standing stocks is linked to the DCM. Thus, as hypothesized, this positioning also appears to influence the foraminiferal ACDs at our study location in the Maldives in the northern equatorial Indian Ocean.

**Fig 11:**
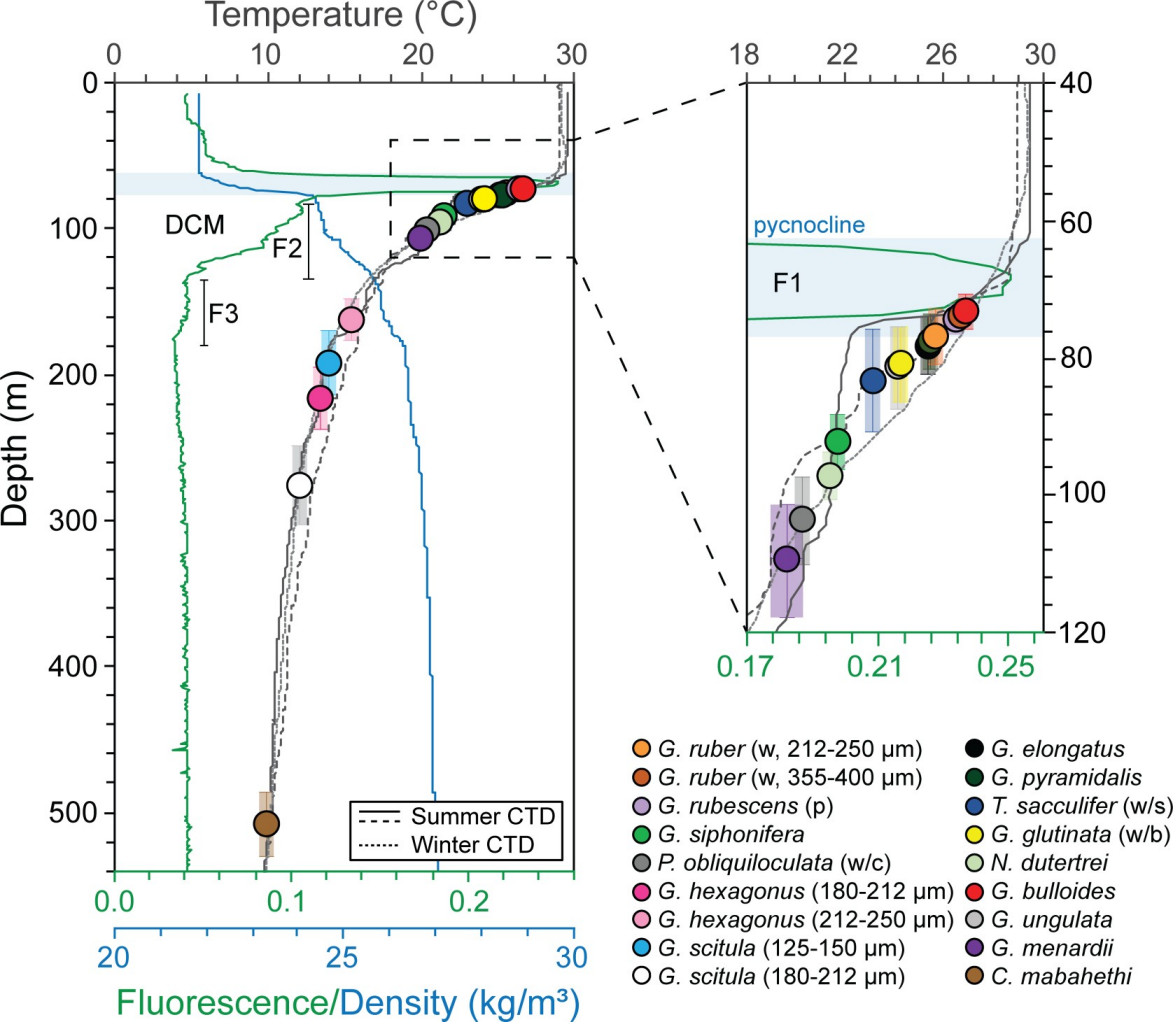
Average isotope-ACDs overlain on the modern day summer (coarsely dashed and solid line) and winter (finely dashed line) CTD data of [24], [26] and [27], respectively. These seasonal CTD datasets were used to show the seasonality and calculate the overall CTD averages. Standard deviations are represented by the colored bars for each species with reference to the regional fluorescence and seawater density profiles also given. DCM: deep chlorophyll maximum with fluorescence peaks F1, F2 and F3; w = white; p = pink; w/s = with sac; w/b = with bulla; w/c = with cortex.

All investigated shallow to intermediate-dwelling species congregate at the base of the SML and in the upper thermocline around the DCM peaks F1 and F2 with an average range between 73–109 m depth (Figs 10 and 11). This DCM is generally situated between the upper nutrient-depleted waters and the lower light depths of the euphotic zone in pelagic oceans. Generally, it is associated with a high production and biomass of phytoplankton [82]. As the geochemical signatures are weighted by the final few precipitated chambers, and we can assume that the majority of the adult specimens measured in this study underwent reproduction, particularly in the case of *G*. *ruber* (w), *G*. *glutinata* (w/b), *G*. *rubescens* (p) and *T*. *sacculifer* (w/s), the observed concentration around the DCM peaks is justified. The DCM thus not only provides a source of prey for adult/pre-adult foraminifera, but the enhanced survival of juveniles is also supported. Additionally, high temperatures in conjunction with high light levels could force the typical surfacing dwelling foraminifera deeper into the water column.

**Fig 12:**
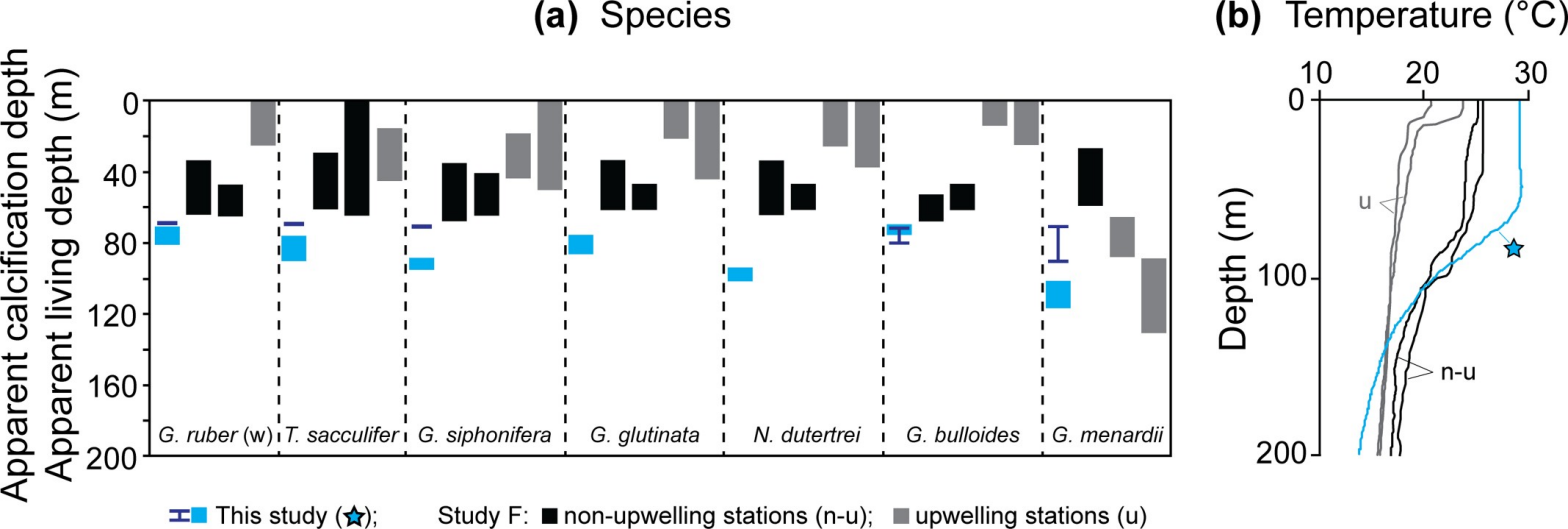
Comparison of the ACDs calculated from this study (Blue blocks: average of the best isotope-ACDs for Method 1.1 and 1.2; dark blue bars denote the Mg/Ca-ACD estimates from Method 2) with the adult ALD estimates from the plankton tows of [3], with their non-upwelling stations represented in black and upwelling stations represented in grey.

As a result of the SAM, the northern Indian Ocean and Arabian Sea have a wide range of biogeochemical provinces. The north-western portions of the Arabian Sea have high productivity and upwelling zones whereas our study site in the Maldives, in the eastern edge, is oligotrophic with little or no upwelling [83]. The closest regional analogue to this study is the depth stratified plankton tow research of [3] from the western Arabian Sea (Study F; Fig 1). With seven species being in common, our calculated ACDs are marginally deeper than their reported ALDs at the non-upwelling stations (Fig 12). These differences could be a result of the geochemical signatures of adult tests being weighted by the final few precipitated chambers, which generally form when they are deeper in the water column, in addition to variations in the vertical structure of the water column. The DCM and SML are positioned shallower in the Western Arabian Sea, between 29–45 m (Fig 12). Furthermore, [3] found two maxima in test concentrations at their non-upwelling sites, the first was at the surface dominated by juveniles with a second deeper maximum linked with the DCM and represented by adult specimens. This is in accordance with the present study, albeit our DCM sits deeper in the water column in comparison to the study of [3].

### Shallow and intermediate-dwelling species

Symbiont-bearing species have a light dependency; making them more prolific in the tropics and sub-tropics within the SML. Five symbiont-bearing species were included in our study: *G*. *ruber* (w), its morphotype *G*. *pyramidalis*, *G*. *elongatus*, *T*. *sacculifer* (w/s) and *G*. *siphonifera*. Furthermore, three additional shallow-dwelling species were analysed *G*. *glutinata* (w/b), *G*. *rubescens* (p) and *N*. *dutertrei*. According to [1,2] both *G*. *glutinata* (w/b) and *N*. *dutertrei* have been found to facultatively host algal symbionts, whereas, *G*. *rubescens* (p) could possibly host symbionts, based on its phylogenetic placement.

All typical SML species ([12] and references within) have shallow ACDs ranging between 74–92 m reflecting their affinity for the photic zone. The shallowest dwellers in this group are the larger *G*. *ruber* specimens and *G*. *rubescens* (p), (both isotope-ACDs = 74±2 m) with the deepest dweller being *G*. *siphonifera* (isotope ACD = 92±4 m).

[17] recognized the close link between *G*. *rube*r (w) and *T*. *sacculifer* (w/s) and the DCM in oligotrophic waters and as shown here even the symbiont-bearing species appear to utilize it as a food source. [17] reported *T*. *sacculifer* (w/s) having a deeper ACD as opposed to *G*. *ruber* in areas with a thick SML, whereas similar depths have been attributed in regions with a shallow SML. Our study, with a shallow local SML, supports this as both *G*. *ruber* (w, 212–250 μm), (isotope-ACD = 77±4 m and Mg/Ca ACD = 70±1 m) and *T*. *sacculifer* (w/s), (isotope-ACD = 83±7 m and Mg/Ca ACD = 70 m) have over-lapping estimates. The species, *G*. *ruber* (w) is generally considered to dwell within the first 30 m of the water column [17] with a preferable temperature around ±27°C [84]. Although our ACD estimates are deeper, they agree well with this optimal temperature. Furthermore, the *G*. *ruber* (w) group is considered to be composed of a number of morphotypes (i.e. *G*. *pyramidalis* and *G*. *elongatus* or *G*. *ruber* sensu stricto and sensu lato) with studies by [85–88] suggesting differing calcification depths for each. Based on the work of [89], *G*. *elongatus* is currently considered a separate species in the modern fauna, as opposed to *G*. *pyramidalis* which is still a morphotype of the classical *G*. *ruber* (w). Our data shows comparable ACDs for all three with identical isotope-ACDs for *G*. *elongatus* and *G*. *pyramidalis* of 78±4 m.

Our ACDs conform to other studies identifying *T*. *sacculifer* as dwelling within the first 80 m of the water column [16,17]. Furthermore, *T*. *sacculifer* has been reported to migrate to deeper depths to reproduce [1,90]. Specimens at the end of their ontogenetic cycle were selected for analysis in this study, based on the assumption that all specimens with a sac have undergone reproduction [91]. Thus, we can assume that our ACD estimates reflect this gametogenic affinity.

With two recognised morphotypes of *G*. *siphonifera* [1,32], we strictly picked the large evolute forms conforming to Type I for the geochemical analyses. Both the isotope- and Mg/Ca-ACD estimates, 92±4 m and 72±1 m respectively, position this species deeper in the water column in the upper thermocline. This is in accordance with observations for the Type I morphotype in the Caribbean [92]. While deeper in the water column, it is still within the photic zone and as such, its symbionts can still be supported.

Both *G*. *glutinata* (w/b) and *G*. *rubescens* (p) are small (<250 μm), ubiquitous species in tropical/subtropical surface waters with large ranges reported in their depth habitats. At the study site *G*. *glutinata* (w/b) lives deeper in the upper thermocline at 81±5 m below the DCM peak F1 at the base of the pycnocline (Fig 11). The latter, *G*. *rubescens* (p) has a marginally shallower isotope-ACD of 74±2 m and according to past studies [2,29,93,94] its vertical distribution is heavily controlled by nutrient availability. Our isotope-ACD estimate places this species in the upper thermocline within peak F1, which would support the premise of it displaying some light dependency, while still being positioned close to the DCM.

*Neogloboquadrina dutertrei* has been shown to facultatively host symbionts [2,29], yet similarly to *G*. *bulloides* is an opportunistic species [95]. Their depth habitats are thus heavily governed by prey availability and local hydrography and consequently both species are recognized as SML/thermocline dwellers. The former, *N*. *dutertrei* has a deeper ACD (isotope-ACD = 97±4 m) than *G*. *bulloides*. *Globigerina bulloides* has the shallowest isotope-ACD estimate of 73±2 m with its Mg/Ca-ACD comparable at 76±4 m. Both these estimates place it within the DCM peak F1, which is surmised to reflect its opportunistic behavior.

*Pulleniatina obliquiloculata* (w/c) and the globorotalids *G*. *menardii* and *G*. *ungulata* are considered to be SML to thermocline dwellers with their highest standing stocks linked to the pcynocline/DCM [1,96,97]. *Globorotalia ungulata* (isotope-ACD = 81±6 m) has an ACD positioned in the upper thermocline whereas both *P*. *obliquiloculata* (w/c), (isotope-ACD = 104±6 m; Mg/Ca-ACD = 74±3 m) and *G*. *menardii* (isotope-ACD = 109±8 m; Mg/Ca-ACD = 70–90 m) are situated in the lower thermocline. The species *P*. *obliquiloculata* (w/c) is known to precipitate gametogenic calcite towards the end of its ontogenetic cycle as it migrates to deeper depths forming a cortex [1]. Specimens used for this study had this smooth layer of pre-gametogenic calcite, thus its ACD is deeper in the upper thermocline close to the DCM peak F2. Similarly, both thermocline-dwelling globorotalids (*G*. *menardii* and *G*. *ungulata*) are associated with the DCM peak F2 with the latter shallower at the base of the pycnocline.

### Deeper-dwelling species

The deepest dwelling species are *G*. *hexagonus* and *G*. *scitula*. *Globorotalia scitula* is cosmopolitan whereas, *G*. *hexagonus* is rare and restricted to the Indo-Pacific [98], however, [99] have questioned this species spatial restriction. Juvenile and (pre)adult specimens of both species were included in this study to allow some comment on their life strategies. Overall, both were found to co-occur at sub-thermocline depths. These concomitant depth habitats were similarly reported for the central Arabian Sea [1,100]. Both species appear to be associated to and respond to peak F3 in the fluorescence vertical profile (Fig 11) and thus by inference chlorophyll. Thus, similarly to [100] we associate their depth habitats to food preferences. Considering the oligotrophic study site, these deeper peaks in phytoplankton would likely serve as a food source. Their tolerance for low-oxygen environments is also confirmed as their depths coincide with the most depleted oxygen levels in the top 500 m of the water column (Fig 3).

Differing accounts exist of the environmental tolerances of *G*. *hexagonus*. [1] report it as having a broad tolerance whereas [99] associate it with restricted environmental preferences. Nevertheless, there is general consensus regarding an association to high nutrient conditions [1,17,101] with [17] surmising it to be herbivorous in nature. Furthermore, [102] identified it as an upwelling indicator for the Oligocene and Miocene with [103] using *G*. *hexagonus* δ^13^C records to reconstruct past eastern equatorial Pacific sub-thermocline water mass contributions. Again, this species nutrient affinity can be confirmed in this study (Fig 11). The smaller juveniles (180–212 μm) have a deeper inferred ACD (isotope-ACD = 218 ± 22 m) in comparison to the pre-adults (isotope-ACD = 163 ± 4 m) with the latter associated to DCM peak F3. This could reflect their surmised reproductive strategy of ascending to shallower depths to reproduce [1], this difference in vertical positioning is, however, not mirrored in the isotopic data of [12].

*Globorotalia scitula* is a medium sized foraminifera (>150 μm) and has been reported in highest abundances during periods of enhanced primary productivity [104]. The smaller juveniles (125–150 μm) have a considerably shallower isotope-ACD of 194±22, within peak F3, in comparison to the larger specimens (180–212 μm) sitting deeper at 277±27 m. This species is also surmised by [1] to ascend to shallower depths to reproduce. As the adults/pre-adults similarly had higher δ^18^O_c_ values in [12], which would also equate to deeper ACDs in comparison to the juveniles, further comment on this reproductive strategy cannot be made.

## Conclusions

While this study is not intended as a review of all foraminiferal ACD calculation methods (isotope and Mg/Ca) and available equations, our comparison does highlight the need to acknowledge these different criteria, in addition to regional hydrological differences, when comparing global studies. Both ACD calculation methods result in variable erroneous values as each requires the selection of suitable equations, the justification of which is not always straightforward. In all instances, the species-specific equations are found to be most robust. However, in their absence a single or paired selection of equations for all species can yield similar results when suitable δ^18^O_sw_ values are applied particularly for thermocline and sub-thermocline dwelling species. Furthermore, while species-isotopic offsets are inherently incorporated, a correction can be applied to account for these disequilibrium effects. We did not apply any corrections in this study, as they were not available for all investigated species. Additionally, applying different δ^18^O-temperature equations for all species affects the absolute ACD values yet the relative species-specific vertical ordering remains consistent. This is an important consideration when comparing data with the published literature. Lastly, while the Mg/Ca-ACD calculation method is not used as the primary approach in this study, the ACDs prove comparable, within 30 m, to the isotope-ACD allocations.

Overall, previously reported planktonic foraminiferal ecological affinities are confirmed for the northern equatorial Indian Ocean for the 14 investigated species. Presently, the water column is highly stratified at the study site in the Maldives, with all ACDs of the shallow- and intermediate-dwellers positioned at the base of the SML and along the thermocline between 73–109 m depth. The DCM appears as a primary control for these shallower-dwelling species, with the sub-thermocline species depth habitats possibly linked to secondary peaks in the local primary production. The shallow-dwelling species *G*. *bulloides*, *G*. *ruber* (w), *G*. *elongatus*, *G*. *pyramidalis* and *G*. *rubescens* (p), are positioned at the base of the SML within the pycnocline within or directly below the DCM peak F1. Whereas, *G*. *glutinata* (w/b), *G*. *ungulata* and *T*. *sacculifer* (w/s) are positioned directly below the pycnocline within the salinity maxima. Conversely, the intermediate-dwelling *G*. *siphonifera*, *N*. *dutertrei*, *P*. *obliquiloculata* (w/c) and *G*. *menardii* have inferred ACDs in the lower thermocline. Changes in the apparent responses between these shallow- (e.g. *G*. *ruber* and *G*. *glutinata*), intermediate- (e.g. *N*. *dutertrei* and *G*. *siphonifera*) and deeper-dwellers (*G*. *scitula* and *G*. *hexagonus*) could ultimately be utilized for regional paleoceanographic reconstructions. As such, by using a combination of foraminiferal proxies (i.e. δ^18^O, δ^13^C and Mg/Ca) for select species from these different depth habitats; past changes (e.g. temperature, salinity, nutrients, chlorophyll) in upper ocean stratification can be constrained and linked back to SAM intensity, variability and associated upwelling.

## Supporting information

S1 Table Compilation of δ^18^O_c_-temperature equations. Bold indicates equations used by studies A, B, D and E (Fig 1). The equations identified in grey shading are the selected species-specific equations used in this study, with bolded-grey shading the criteria the selection of the equation was based on.
(DOCX)

S2 Table Compilation of species-specific foraminiferal Mg/Ca-temperature equations. The equations identified in grey shading are the selected species-specific equations used in this study, with bold text indicating the criteria the selection of the equation was based on. Equations with ° and °° were excluded as the calculated temperatures were outside the regional temperature range for planktonic and benthic species, respectively.
(DOCX)
