# Supplementary material for: Controls on planktonic foraminifera apparent calcification depths for the northern equatorial Indian Ocean
Source: PLoS One. 2019 Sep 12;14(9):e0222299. doi: 10.1371/journal.pone.0222299 (PMC6767952; doi:10.1371/journal.pone.0222299)
Supplement: S1 Table — Bold indicates equations used by studies A, B, D and E (Fig 1). The equations identified in grey shading are the selected species-specific equations used in this study, with bolded-grey shading the criteria the selection of the equation was based on. (DOCX) [file pone.0222299.s001.docx]

**S1: Compilation of δ^18^Oc-temperature equations.** Bold indicates equations used by studies A, B, D and E (Fig 1). The equations identified in grey shading are the selected species-specific equations used in this study, with bolded-grey shading the criteria the selection of the equation was based on.

| Reference | Source | Size (μm) | Sample Type | Geographical location | TR (°C) | T (°C) = a + b (δ^18^Oc- δ^18^Osw) + c (δ^18^Oc- δ^18^Osw)^2^ | | |
| --- | --- | --- | --- | --- | --- | --- | --- | --- |
|  |  |  |  |  |  | **a** | **b** | **c** |
| [1] | Synthetic calcite |  |  |  | 0-500 | **16.90** | **-4.38** | **0.10** |
| [2] | Inorganic calcite | N/A | E |  | 10-40 | **16.10** | **-4.64** | **0.09** |
| [3] | Multispecies |  | PT |  |  | **14.32** | **-4.28** | **0.07** |
| [4] | *G. ruber (w)* |  | PT | **Indian Ocean** | **±20-31** | 12.75 | -5.00 |  |
| [5] |  | >150 | PT | Global | ±15.2-30.2 | 14.20 | -4.44 |  |
| [6] |  | 355-425 | CT | Atlantic Ocean |  | 15.40 | -4.78 |  |
| [4] | *T. sacculifer* |  | PT | **Indian Ocean** | **±22.5-31** | 11.95 | -5.26 |  |
| [7] |  | ±150 | CL | Northern Gulf of Eilat | 14-30 | **16.99** | **-4.52** | **0.03** |
| [5] |  | >150 | PT | Global | ±15.2-30.2 | 14.91 | -4.35 |  |
| [8] |  |  | CL |  |  | 12.00 | -5.67 |  |
| [6] |  | 355-425 | CT | Atlantic Ocean |  | 16.20 | -4.94 |  |
| [9] | *N. dutertrei* |  | PT | **Indian Ocean** | **20-29** | 10.50 | -6.58 |  |
| [6] |  | 500-600 | CT | Atlantic Ocean |  | 14.60 | -5.09 |  |
| [10] | ** G. bulloides* | 301 ± 25 | CL | Southern California Bight | 15-24 | 12.60 | -5.07 |  |
| [10] | **** | 369 ± 30 | CL | Southern California Bight | 15-24 | 13.20 | -4.89 |  |
| [10] | ***** | 414 ± 39 | CL | Southern California Bight | 15-24 | 13.60 | -4.77 |  |
| [11] |  |  | PT | **Western Arabian Sea** |  | 14.20 | -4.81 |  |
| [5] |  | >150 | PT | Global | ±0.2-25.2 | 14.62 | -4.70 |  |
| [6] | *P. oblicuiloculata* | 500-600 | CT | Atlantic Ocean |  | 16.80 | -5.22 |  |
| [9] | *G. menardii* | 355-500 | PT | **Indian Ocean** | **20-29** | 14.60 | -5.03 |  |
| [12] |  |  | CL | Indian Ocean |  | 14.90 | -5.13 |  |
| [6] |  | 600-710 | CT | Atlantic Ocean |  | 16.60 | -5.20 |  |
| [13] | *Cibicidoides* & *Planulina* |  |  | **Little Bahama Bank** | **4-26** | 16.09 | -4.76 |  |
| *** (11 chambered shell). **** (12 chambered shell). ***** (13 chambered shell). E: experiment; PT: plankton tow; CT: core top; CL: culture; TR: temperature calibration range | | | | | | | | |

# References

1. Shackleton NJ. Attainment of isotopic equilibrium between ocean water and the benthonic foraminifera Genus *Uvigerina*: Isotopic changes in the ocean during the last glacial. Colloq Int du CNRS. 1974;219: 203–210.

2. Kim S-T, O’Neil JR. Equilibrium and nonequilibrium oxygen isotope effects in synthetic carbonates. Geochim Cosmochim Acta. 1997;61: 3461–3475. doi:10.1016/S0016-7037(97)00169-5

3. Mulitza S, Donner B, Fischer G, Paul A, Pätzold J, Rühlemann C, et al. The South Atlantic Oxygen Isotope Record of Planktonic Foraminifera. In: Wefer G, Mulitza S, Ratmeyer V, editors. The South Atlantic in the Late Quaternary: Reconstruction of Material Budgets and Current Systems. Berlin: Springer; 2004. pp. 121–142.

4. Duplessy JC, Bé AWH, Blanc PL. Oxygen and carbon isotopic composition and biogeographic distribution of planktonic foraminifera in the Indian Ocean. Palaeogeogr Palaeoclimatol Palaeoecol. 1981;33: 9–46. doi:10.1016/0031-0182(81)90031-6

5. Mulitza S, Boltovskoy D, Donner B, Meggers H, Paul A, Wefer G. Temperature: δ^18^O relationships of planktonic foraminifera collected from surface waters. Palaeogeogr Palaeoclimatol Palaeoecol. 2003;202: 143–152. doi:10.1016/S0031-0182(03)00633-3

6. Farmer CE, Kaplan A, de Menocal PB, Lynch-Stieglitz J. Corroborating ecological depth preferences of planktonic foraminifera in the tropical Atlantic with the stable oxygen isotope ratios of core top specimens. Paleoceanography. 2007;22: PA3205. doi:10.1029/2006PA001361

7. Erez J, Luz B. Experimental paleotemperature equation for planktonic foraminifera. Geochim Cosmochim Acta. 1983;47: 1025–1031. doi:10.1016/0016-7037(83)90232-6

8. Spero HJ, Mielke KM, Kalve EM, Lea DW, Pak DK. Multispecies approach to reconstructing eastern equatorial Pacific thermocline hydrography during the past 360 kyr. Paleoceanography. 2003;18: 1022. doi:10.1029/2002PA000814

9. Bouvier-Soumagnac Y, Duplessy J-C. Carbon and oxygen isotopic composition of planktonic foraminifera from laboratory culture, plankton tows and recent sediment; implications for the reconstruction of paleoclimatic conditions and of the global carbon cycle. J Foraminifer Res. 1985;15: 302–320. doi:10.2113/gsjfr.15.4.302

10. Bemis BE, Spero HJ, Bijma J, Lea DW. Reevaluation of the oxygen isotopic composition of planktonic foraminifera: Experimental results and revised paleotemperature equations. Paleoceanography. 1998;13: 150–160. doi:10.1029/98PA00070

11. Peeters FJC. The distribution and stable isotope composition of living planktic foraminifera in relation to seasonal changes in the Arabian Sea. Free University, Amsterdam, The Netherlands. 2000.

12. Mielke KM. Reconstructing Surface Carbonate Chemistry and Temperature in Paleoceans: Geochemical results from laboratory experiments and the fossil record. University of Cardiff. 2001.

13. Lynch-Stieglitz J, Curry WB, Slowey N. A geostrophic transport estimate for the Florida Current from the oxygen isotope composition of benthic foraminifera. Paleoceanography. 1999;14: 360–373. doi:10.1029/1999PA900001
