# Supplementary material for: Controls on planktonic foraminifera apparent calcification depths for the northern equatorial Indian Ocean
Source: PLoS One. 2019 Sep 12;14(9):e0222299. doi: 10.1371/journal.pone.0222299 (PMC6767952; doi:10.1371/journal.pone.0222299)
Supplement: S2 Table — The equations identified in grey shading are the selected species-specific equations used in this study, with bold text indicating the criteria the selection of the equation was based on. Equations with ° and °° were excluded as the calculated temperatures were outside the regional temperature range for planktonic and benthic species, respectively. (DOCX) [file pone.0222299.s002.docx]

**S2: Compilation of species-specific foraminiferal Mg/Ca-temperature equations.** The equations identified in grey shading are the selected species-specific equations used in this study, with bold text indicating the criteria the selection of the equation was based on. Equations with ° and °° were excluded as the calculated temperatures were outside the regional temperature range for planktonic and benthic species respectively.

| Reference | Species | Size (µm) | Sample type | Geographical location | TR (°C) | CM | Mg/Ca = B exp (A*Temperature) | | |
| --- | --- | --- | --- | --- | --- | --- | --- | --- | --- |
|  |  |  |  |  |  |  | **B** | **A** | **R^2^** |
| [1] | *G. ruber* (w) |  | CT | North Atlantic Ocean | ±17-20 |  | 0.52 | 0.100 |  |
| [2]° |  | 250– 350 | CT | Equatorial Pacific Ocean | 23-29 | ID | 0.30 | 0.089 | 0.95 |
| [3]° |  | 250– 350 | CT | Pacific & Atlantic Ocean | 21-29 |  | 0.38 | 0.090 | 0.70 |
| [4] ° |  |  |  |  |  |  | 0.57 | 0.074 |  |
| [5] |  | **250-350** | ST | Sargasso Sea | **16-27** | **O** | 0.34 | 0.102 | 0.91 |
| [5] |  | 350-500 | ST | Sargasso Sea | 16-27 | O | 0.48 | 0.085 | 0.94 |
| [6]° |  | all data | ST | Gulf of California | 20-33 | O | 0.68 | 0.069 | 0.83 |
| [6]° |  | 212-355 | ST | Gulf of California | 20-33 | O | 0.69 | 0.068 | 0.86 |
| [6]° |  | 212-300 | ST | Gulf of California | 20-33 | O | 0.64 | 0.071 | 0.63 |
| [6]° |  | 300-355 | ST | Gulf of California | 20-33 | O | 0.58 | 0.074 | 0.75 |
| [7] |  | 250-315 | CT | North Atlantic | 16.8-28.4 | O | 0.76 | 0.070 | 0.93 |
| [8]° |  | > 250 |  | Eastern Indian Ocean | 18.5-29.2 | US | 0.52 | 0.076 | 0.99 |
| [9] |  | 355–400 | SS | Atlantic & Caribbean | 24–28 | O | 0.40 | 0.094 | 0.72 |
| [10] ° |  | 250-355 | SS | Western Pacific Warm Pool | 28-30 | R | 0.31 | 0.097 |  |
| [11]* |  |  |  |  | 22–28 |  | 1.28 | 0.051 | 0.53 |
| [12]* |  |  |  |  | 21.5–28 |  | 1.51 | 0.045 | 0.52 |
| [13]* |  |  |  |  | 22–28 |  | 1.27 | 0.051 | 0.54 |
| [14]* |  |  |  |  | 20–27.5 |  | 2.04 | 0.034 | 0.49 |
| [15]* |  |  |  |  | 19–26.5 |  | 2.32 | 0.031 | 0.48 |
| [16]* |  |  |  |  | 19–27 |  | 2.32 | 0.031 | 0.49 |
| [17]* |  |  |  |  | 20.5–28 |  | 1.87 | 0.037 | 0.50 |
| [18] | *T. sacculifer* | 110-500 | CL |  | 19.5-29.5 |  | 0.39 | 0.089 |  |
| [1] |  |  | CT | North Atlantic Ocean | ±17-20 |  | 0.50 | 0.100 |  |
| [19]° |  | 250-500 | C | Tropical Atlantic Ocean |  |  | 0.49 | 0.076 |  |
| [3] | without sac | 250– 350 | CT | Pacific & Atlantic Ocean | ±22-28 |  | 0.37 | 0.090 | 0.67 |
| [5]° | without sac | 350-500 | ST | Sargasso Sea | 16-27 | O | 1.06 | 0.048 | 0.75 |
| [5] | with sac | 350-500 | ST | Sargasso Sea | 16-27 | O | 0.67 | 0.069 | 0.85 |
| [9] |  | **355–400** | SS | Atlantic Ocean & Caribbean | **23-27** | **O** | 0.60 | 0.075 | 0.80 |
| [10]° | with sac | 250-355 | SS | Western Pacific Warm Pool | 27.5-30 | R | 0.24 | 0.097 |  |
| [11]* |  |  |  |  | 23–27.5 |  | 0.44 | 0.086 | 0.77 |
| [20]* |  |  |  |  | 23–27.5 |  | 0.40 | 0.089 | 0.77 |
| [12]* |  |  |  |  | 22.5–27.5 |  | 0.52 | 0.080 | 0.77 |
| [13]* |  |  |  |  | 23–27.5 |  | 0.46 | 0.083 | 0.76 |
| [15]* |  |  |  |  | 20.5–26 |  | 0.64 | 0.077 | 0.78 |
| [21]* |  |  |  |  | 19.5–26.5 |  | 0.98 | 0.060 | 0.78 |
| [16]* |  |  |  |  | 20–26. 0 |  | 0.74 | 0.072 | 0.77 |
| [17]* |  |  |  |  | 22.5–27.5 |  | 0.47 | 0.083 | 0.79 |
| [1] | *G. siphonifera* |  | CT | North Atlantic Ocean | 8-22 |  | 0.52 | 0.100 |  |
| [5] |  | 350-500 | ST | North Atlantic Ocean | 16-27 | **O** | 0.86 | 0.068 | 0.67 |
| [22] | *G. bulloides* |  | CL | San Pedro Basin | **16-25** |  | 0.53 | 0.102 | 0.93 |
| [23] |  |  | CL, CT | Subantarctic Indian Ocean | ±10-25 |  | 0.47 | 0.107 | 0.98 |
| [1] |  |  | CT | North Atlantic Ocean | ±8-15 |  | 0.56 | 0.100 |  |
| [6]° |  | all data | ST | Gulf of California | 16-31 | O | 1.23 | 0.057 | 0.88 |
| [6]° |  | 212-355 | ST | Gulf of California | 16-31 | O | 1.20 | 0.057 | 0.90 |
| [6]° |  | 212-300 | ST | Gulf of California | 16-31 | O | 1.53 | 0.049 | 0.81 |
| [6]° |  | 300-355 | ST | Gulf of California | 16-31 | O | 1.03 | 0.064 | 0.87 |
| [7] |  | 250-315 | CT | North Atlantic Ocean | 10.3-17.6 | O | 0.78 | 0.082 | 0.87 |
| [5] | *P. obliquiloculata* | **350-500** | ST | Sargasso Sea | **16-27** | **O** | 0.18 | 0.120 | 0.79 |
| [7]° |  | 355-400 | CT | North Atlantic Ocean | 20-25.2 | O | 1.02 | 0.039 |  |
| [10]° |  | 355-425 | SS | Western Pacific Warm Pool | 24.5-30 | R | 0.21 | 0.097 |  |
| [9] | *G. menardii* | **250–500** | SS | Atlantic & Caribbean | **21-26** | **O** | 0.36 | 0.091 | 0.66 |
| [11]* |  |  |  |  | 17.5–27 |  | 0.86 | 0.057 | 0.72 |
| [24]* |  |  |  |  | 13.5–26.5 |  | 1.17 | 0.047 | 0.72 |
| [12]* |  |  |  |  | 16.5–27 |  | 0.97 | 0.053 | 0.72 |
| [13]* |  |  |  |  | 17–27 |  | 0.91 | 0.055 | 0.73 |
| [25]* |  |  |  |  | 14.5–26.5 |  | 1.13 | 0.048 | 0.72 |
| [16]* |  |  |  |  | 13.5–25.5 |  | 1.18 | 0.050 | 0.70 |
| [17]*° |  |  |  |  | 17–27 |  | 0.93 | 0.052 | 0.73 |
| [26]°° | *Cibicidoides* spp. | >250 | CT | Global | 0.8–18.4 | R | 0.87 | 0.109 |  |
| [27]°° |  |  | CT | Tropical Atlantic and Pacific | −1.1–18 | R | 1.22 | 0.109 |  |
| [28] |  |  | CT | Global | −1.1–18 | O | 0.90 | 0.110 |  |
| [29]°° | *C. wuellerstorfi* |  | CT | Global | 0.95–3.8 | R | 0.781 | 0.230 | 0.91 |
| [30] |  | >250 | CT | South Atlantic Ocean | 0-15 |  | 0.83 | 0.145 |  |
| [31]°° |  |  | CT, C | North Atlantic Ocean | 0.98–4.26 | O | 0.59 | 0.280 |  |
| [32]°° |  | >150 | CT | Tropical Atlantic Ocean | 0–6 | O | 0.82 | 0.190 | 0.73 |
| [33] |  |  | CT | Indian and Pacific Ocean | **0-10** | R | 0.71 | 0.150 | 0.68 |
| [34]°° |  | >250 | CT, SS | North Pacific Ocean | 1.7–16.3 | R | 0.85 | 0.082 | 0.97 |
| C: Cores; CT: Core top; ST: sediment trap; SS: surface sediment; CL: culture; TR: temperature range; CM: cleaning method; O: oxidative; R: reductive; ID: isotope dilution; US: ultra sound. ° indicates equations which yielded calculated temperatures for planktonic species above the maximum recorded in the study region; °° indicates equations which yielded calculated temperatures for benthic species outside the range for the depth of the study site and * denotes equations calibrated by [9] using the referenced δ^18^O_c_-temperature equation | | | | | | | | | |

# References

1. Elderfield H, Ganssen G. Past temperature and δ^18^O of surface ocean waters inferred from foraminiferal Mg/Ca ratios. Nature. 2000;405: 442–445. doi:10.1038/35013033

2. Lea DW, Pak DK, Spero HJ. Climate Impact of Late Quaternary Equatorial Pacific Sea Surface Temperature Variations. Science (80). 2000;289: 1719–1724. doi:10.1126/science.289.5485.1719

3. Dekens PS, Lea DW, Pak DK, Spero HJ. Core top calibration of Mg/Ca in tropical foraminifera: Refining paleotemperature estimation. Geochemistry, Geophys Geosystems. 2002;3. doi:10.1029/2001GC000200

4. Whitko AN, Hastings DW, Flower BP. Past sea surface temperatures in the tropical South China Sea based on a new foraminiferal Mg calibration. MARsci. 2002; doi:MARSci.2002.01.020101.

5. Anand P, Elderfield H, Conte MH. Calibration of Mg/Ca thermometry in planktonic foraminifera from a sediment trap time series. Paleoceanography. 2003;18: 1050. doi:10.1029/2002PA000846

6. McConnell MC, Thunell RC. Calibration of the planktonic foraminiferal Mg/Ca paleothermometer: Sediment trap results from the Guaymas Basin, Gulf of California. Paleoceanography. 2005;20: PA2016. doi:10.1029/2004PA001077

7. Cléroux C, Cortijo E, Anand P, Labeyrie L, Bassinot F, Caillon N, et al. Mg/Ca and Sr/Ca ratios in planktonic foraminifera: Proxies for upper water column temperature reconstruction. Paleoceanography. 2008;23: PA3214. doi:10.1029/2007PA001505

8. Sadekov A, Eggins SM, De Deckker P, Kroon D. Uncertainties in seawater thermometry deriving from intratest and intertest Mg/Ca variability in *Globigerinoides ruber*. Paleoceanography. 2008;23: PA1215. doi:10.1029/2007PA001452

9. Regenberg M, Steph S, Nürnberg D, Tiedemann R, Garbe-Schönberg D. Calibrating Mg/Ca ratios of multiple planktonic foraminiferal species with δ^18^O-calcification temperatures: Paleothermometry for the upper water column. Earth Planet Sci Lett. Elsevier B.V.; 2009;278: 324–336. doi:10.1016/j.epsl.2008.12.019

10. Hollstein M, Mohtadi M, Rosenthal Y, Moffa Sanchez P, Oppo D, Martínez Méndez G, et al. Stable Oxygen Isotopes and Mg/Ca in Planktic Foraminifera From Modern Surface Sediments of the Western Pacific Warm Pool: Implications for Thermocline Reconstructions. Paleoceanography. 2017;32: 1174–1194. doi:10.1002/2017PA003122

11. Shackleton NJ. Attainment of isotopic equilibrium between ocean water and the benthonic foraminifera Genus *Uvigerina*: Isotopic changes in the ocean during the last glacial. Colloq Int du CNRS. 1974;219: 203–210.

12. Kim S-T, O’Neil JR. Equilibrium and nonequilibrium oxygen isotope effects in synthetic carbonates. Geochim Cosmochim Acta. 1997;61: 3461–3475. doi:10.1016/S0016-7037(97)00169-5

13. Bemis BE, Spero HJ, Bijma J, Lea DW. Reevaluation of the oxygen isotopic composition of planktonic foraminifera: Experimental results and revised paleotemperature equations. Paleoceanography. 1998;13: 150–160. doi:10.1029/98PA00070

14. Thunell R, Tappa E, Pride C, Kincaid E. Sea-surface temperature anomalies associated with the 1997–1998 El Niño recorded in the oxygen isotope composition of planktonic foraminifera. Geology. 1999;27: 843. doi:10.1130/0091-7613(1999)027<0843:SSTAAW>2.3.CO;2

15. Mulitza S, Boltovskoy D, Donner B, Meggers H, Paul A, Wefer G. Temperature: δ^18^O relationships of planktonic foraminifera collected from surface waters. Palaeogeogr Palaeoclimatol Palaeoecol. 2003;202: 143–152. doi:10.1016/S0031-0182(03)00633-3

16. Mulitza S, Donner B, Fischer G, Paul A, Pätzold J, Rühlemann C, et al. The South Atlantic Oxygen Isotope Record of Planktonic Foraminifera. In: Wefer G, Mulitza S, Ratmeyer V, editors. The South Atlantic in the Late Quaternary: Reconstruction of Material Budgets and Current Systems. Berlin: Springer; 2004. pp. 121–142.

17. Farmer CE, Kaplan A, de Menocal PB, Lynch-Stieglitz J. Corroborating ecological depth preferences of planktonic foraminifera in the tropical Atlantic with the stable oxygen isotope ratios of core top specimens. Paleoceanography. 2007;22: PA3205. doi:10.1029/2006PA001361

18. Nürnberg D, Bijma J, Hemleben C. Assessing the reliability of magnesium in foraminiferal calcite as a proxy for water mass temperatures. Geochim Cosmochim Acta. 1996;60: 803–814. doi:10.1016/0016-7037(95)00446-7

19. Nürnberg D, Müller A, Schneider RR. Paleo-sea surface temperature calculations in the equatorial east Atlantic from Mg/Ca ratios in planktic foraminifera: A comparison to sea surface temperature estimates from U_37_^K^, oxygen isotopes, and foraminiferal transfer function. Paleoceanography. John Wiley & Sons, Ltd; 2000;15: 124–134. doi:10.1029/1999PA000370

20. Erez J, Luz B. Experimental paleotemperature equation for planktonic foraminifera. Geochim Cosmochim Acta. 1983;47: 1025–1031. doi:10.1016/0016-7037(83)90232-6

21. Spero HJ, Mielke KM, Kalve EM, Lea DW, Pak DK. Multispecies approach to reconstructing eastern equatorial Pacific thermocline hydrography during the past 360 kyr. Paleoceanography. 2003;18: 1022. doi:10.1029/2002PA000814

22. Lea DW, Mashiotta TA, Spero HJ. Controls on magnesium and strontium uptake in planktonic foraminifera determined by live culturing. Geochim Cosmochim Acta. 1999;63: 2369–2379. doi:10.1016/S0016-7037(99)00197-0

23. Mashiotta TA, Lea DW, Spero HJ. Glacial–interglacial changes in Subantarctic sea surface temperature and δ^18^O-water using foraminiferal Mg. Earth Planet Sci Lett. Elsevier; 1999;170: 417–432. doi:10.1016/S0012-821X(99)00116-8

24. Bouvier-Soumagnac Y, Duplessy J-C. Carbon and oxygen isotopic composition of planktonic foraminifera from laboratory culture, plankton tows and recent sediment; implications for the reconstruction of paleoclimatic conditions and of the global carbon cycle. J Foraminifer Res. 1985;15: 302–320. doi:10.2113/gsjfr.15.4.302

25. Mielke KM. Reconstructing Surface Carbonate Chemistry and Temperature in Paleoceans: Geochemical results from laboratory experiments and the fossil record. University of Cardiff. 2001.

26. Lear CH, Rosenthal Y, Slowey N. Benthic foraminiferal Mg/Ca paleothermometry: A revised core-top calibration. Geochim Cosmochim Acta. 2002;66: 3375–3387.

27. Martin PA, Lea DW, Rosenthal Y, Shackleton NJ, Sarnthein M, Papenfuss T. Quaternary deep sea temperature histories derived from benthic foraminiferal Mg/Ca. Earth Planet Sci Lett. 2002;198: 193–209. doi:10.1016/S0012-821X(02)00472-7

28. Elderfield H, Yu J, Anand P, Kiefer T, Nyland B. Calibrations for benthic foraminiferal Mg/Ca paleothermometry and the carbonate ion hypothesis. Earth Planet Sci Lett. 2006;250: 633–649. doi:doi:10.1016/j.epsl.2006.07.041

29. Healey SL, Thunell RC, Corliss BH. The Mg/Ca-temperature relationship of benthic foraminiferal calcite: New core-top calibrations in the < 4 °C temperature range. Earth Planet Sci Lett. 2008;272: 523–530. doi:10.1016/j.epsl.2008.05.023

30. Raitzsch M, Kuhnert H, Groeneveld J, Bickert T. Benthic foraminifer Mg/Ca anomalies in South Atlantic core top sediments and their implications for paleothermometry. Geochemistry, Geophys Geosystems. John Wiley & Sons, Ltd; 2008;9: Q05010. doi:10.1029/2007GC001788

31. Yu J, Elderfield H. Mg/Ca in the benthic foraminifera *Cibicidoides wuellerstorfi* and *Cibicidoides mundulus*: Temperature versus carbonate ion saturation. Earth Planet Sci Lett. 2008;276: 129–139. doi:10.1016/j.epsl.2008.09.015

32. Tisserand AA, Dokken TM, Waelbroeck C, Gherardi J-M, Scao V, Fontanier C, et al. Refining benthic foraminiferal Mg/Ca-temperature calibrations using core-tops from the western tropical Atlantic: Implication for paleotemperature estimation. Geochemistry, Geophys Geosystems. John Wiley & Sons, Ltd; 2013;14: 929–946. doi:10.1002/ggge.20043

33. Lo Giudice Cappelli E, Regenberg M, Holbourn A, Kuhnt W, Garbe-schönberg D, Andersen N. Refining *C. wuellerstorfi* and *H. elegans* Mg/Ca temperature calibrations. Mar Micropaleontol. 2015;121: 70–84. doi:org/10.1016/j.marmicro.2015.10.001 0377-8398/©

34. Kubota Y, Kimoto K, Itaki T, Yokoyama Y, Miyairi Y, Matsuzaki H. Bottom water variability in the subtropical northwestern Pacific from 26 kyr BP to present based on Mg / Ca and stable carbon and oxygen isotopes of benthic foraminifera. Clim Past. 2015;11: 803–824. doi:10.5194/cp-11-803-2015
